# Steric and electronic effects on the ^1^H hyperpolarisation of substituted pyridazines by signal amplification by reversible exchange

**DOI:** 10.1002/mrc.5152

**Published:** 2021-04-05

**Authors:** Peter J. Rayner, Michael J. Burns, Elizabeth J. Fear, Simon B. Duckett

**Affiliations:** ^1^ Centre for Hyperpolarisation in Magnetic Resonance, Department of Chemistry University of York York UK

**Keywords:** hyperpolarisation, NMR, parahydrogen, SABRE

## Abstract

Utility of the pyridazine motif is growing in popularity as pharmaceutical and agrochemical agents. The detection and structural characterisation of such materials is therefore imperative for the successful development of new products. Signal amplification by reversible exchange (SABRE) offers a route to dramatically improve the sensitivity of magnetic resonance methods, and we apply it here to the rapid and cost‐effective hyperpolarisation of substituted pyridazines. The 33 substrates investigated cover a range of steric and electronic properties and their capacity to perform highly effective SABRE is assessed. We find the method to be tolerant to a broad range of electron donating and withdrawing groups; however, good sensitivity is evident when steric bulk is added to the 3‐ and 6‐positions of the pyridazine ring. We optimise the method by reference to a disubstituted ester that yields signal gains of >9000‐fold at 9.4 T (>28% spin polarisation).

## Graphical abstract

SABRE enables pyridazines to be detected by ^1^H NMR with greater than 28% polarisation.

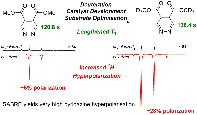

## 1 INTRODUCTION

Pyridazine is a dinitrogen containing aromatic heterocycle that contains two *sp* ^*2*^‐hybridised nitrogen atoms within a six‐membered ring. Whilst being isosteric with benzene, it offers increased possibilities for molecular interactions, higher electron density, lower LogP values and improved crystallinity. This has resulted in its use increasing in both pharmaceutical and agrochemical industries.^[^ ^1^ ^]^ Indeed, the replacement of phenyl rings within drug molecules has led to several hundred articles dealing with bioactive pyridazines that belong to almost all therapeutic classes in the last 15 years alone.^[^ ^2^ ^]^ As such, pyridazine has been described as a ‘privileged structure’ within the field of medicinal chemistry.^[^ ^3^ ^]^ Most recent examples have focused on the synthesis and evaluation of fused *bi‐* and *tri‐*cyclic pyridazines such as imidazo,^[^ ^2^ ^]^ pyrrolo^[^ ^4^ ^]^ and thienopyridazines.^[^ ^5^ ^]^

With such prevalence of pyridazines in medicinal and agricultural chemistry, routes to their rapid and accurate detection and structural determination are necessary. Nuclear magnetic resonance (NMR) is one of the most widely used methods in the analytic sciences and offers unique insight into molecular structure, activity and environment. However, when compared with other analytical techniques, such as mass spectrometry or infrared spectroscopy, the required sample size and concentration are much higher. This is due to the lower sensitivity of NMR that is caused by very small population differences between the magnetic state energy levels that the method probes. Classically, this insensitivity is overcome by signal averaging and increased concentrations, which has time and cost implications. However, this sensitivity challenge will hinder the ability of NMR to rapidly detect transient intermediates or trace impurities.

NMR hyperpolarisation offers an attractive solution by creating magnetic state energy level populations that deviate from the Boltzmann distribution.^[^ ^6^ ^]^ This has led to the development of methods with sensitivity gains that are many orders of magnitude higher than could be achieved using conventional NMR. These large signals have been widely exploited for probing reaction mechanisms,^[^ ^7^ ^]^ detection of low concentration species^[^ ^8^ ^]^ and the in vivo quantification of anatomy^[^ ^9^ ^]^ and metabolism^[^ ^10^ ^]^ amongst others. A number of methods for creating the hyperpolarised species are known with spin exchange optical pumping (SEOP),^[^ ^11^ ^]^ dynamic nuclear polarisation (DNP)^[^ ^6^, ^12^ ^]^ and *para*hydrogen induced polarisation (PHIP)^[^ ^6^, ^13^ ^]^ being the most common.

Signal amplification by reversible exchange (SABRE)^[^ ^14^ ^]^ is a PHIP method that derives its hyperpolarised spin order from the singlet state of hydrogen, *para*hydrogen (*p*‐H_2_). SABRE typically utilises an iridium *N*‐heterocyclic catalyst^[^ ^15^ ^]^ that reversible binds *p*‐H_2_, as hydride ligands, and a substrate molecule such that spin polarisation is transferred through the temporarily formed *J*‐coupling network.^[^ ^16^ ^]^ Since its first report in 2009, SABRE has received wide attention from the research community, and the method and its applications have been recently reviewed in detail.^[^ ^17^ ^]^

Pyridazine has previously been shown to be amenable to SABRE hyperpolarisation, and the mechanism for the formation of the active SABRE catalyst is well defined.^[^ ^18^ ^]^ When [IrCl (COD)(IMes)] (where COD = 1,5‐cyclooctadience and IMes = 1,3‐bis(2,4,6‐trimethylphenyl)imidazol‐2‐ylidene) is reacted with H_2_ in the presence of pyridazine at 298 K, [Ir(H)_2_(IMes)(pyridazine)_3_]Cl is formed which undergoes reversible ligand exchange in methanol‐*d*_4_. Additionally, a hapotropic shift between the two nitrogen atoms in the ring is also observed using EXSY. When this sample was exposed to *p*‐H_2_ (3 bar), a ca. 400‐fold ^1^H signal gain was observed at 9.4 T for its two proton resonances after polarisation transfer at 65 G. The use of selective deuteration enabled the SABRE polarisation level to increase such that 3,5‐*d*_2_‐pyridazine yields polarisation levels of greater than 22% (>7000‐fold signal gain at 9.4 T) in conjunction with *T*_1_ lifetimes that approach 3 min when a deuterated catalyst and 5 bar pressure of *p*‐H_2_ were employed.^[^ ^19^ ^]^ The high symmetry of the pyridazine motif has also been exploited for the creation of long‐lived magnetic states that allow detection of a ^1^H NMR signal 15 min after its creation.^[^ ^20^ ^]^ Additionally, SABRE hyperpolarisation of its ^13^C and ^15^N nuclei with in pyridazine has also been reported.^[^ ^21^ ^]^

Here, we wish to report the effects of pyridazine functional group substitution on the SABRE derived ^1^H NMR enhancements and *T*_1_ relaxation lifetimes. Over 30 mono and disubstituted pyridazines are investigated that encompass a range of steric and electronic properties in this study. This allows us to draw conclusions as to what are the optimum substitution patterns, in addition to defining the functional group tolerance on the SABRE effect.

## 2 RESULTS AND DISCUSSION

Our examination of the effects of pyridazine substitution began with the study of mono substituted substrates prior to building in complexity. The 3‐Substituted pyridazines **2**–**7** shown in Table 1 were purchased or synthesised as described in the supporting information. NMR tubes equipped with a *J*. Young's Tap were filled with a solution containing [IrCl (COD)(IMes)] (5 mM), substrate (20 mM) in methanol‐*d*_4_ and exposed to H_2_ (3 bar). After 1 h at room temperature, the samples were analysed by ^1^H NMR spectroscopy for the formation of new hydride containing complexes. For comparison, we also recorded the ^1^H NMR spectrum of a sample containing pyridazine **1**, which showed a single hydride containing complex with a resonance at *δ*_H_ –21.49 that is consistent with [Ir(H)_2_(IMes)(pyridazine)_3_]Cl as previously reported in the literature.^[^ ^18^ ^]^ The headspace of this sample is then replaced with 3 bar *p*‐H_2_ (>99% enrichment) and manually shaken in a 70 G polarisation transfer field, prior to rapid insertion into a 9.4 T NMR spectrometer and a ^1^H NMR spectrum was immediately recorded (full experimental details are available in the supporting information). This ^1^H NMR spectrum showed per proton signal enhancements of 316‐ and 472‐fold for the *ortho* and *meta* resonances of pyridazine after SABRE polarisation transfer at 70 G after quantification. *T*_1_ relaxation values of 29.1 s for the *ortho* (H3/6) resonance and 26.6 s for the *meta* (H4/5) resonance were observed for this sample.

**TABLE 1:** Summary of SABRE hyperpolarisation and *T*_1_ relaxation times of 3‐substituted pyridazines

Substrate	Hydride resonance for [Ir(H)_2_(IMes)(sub)_3_]cl at 298 K/ppm	^1^H SABRE signal gain at 9.4 T (% polarisation)	*T*_1_ relaxation time at 298 K measured at 9.4 T/s
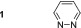	−21.49	H3/6‐472 ± 24 (1.5%)	H3/6‐29.1
		H4/5‐316 ± 42 (1.0%)	H4/5‐26.6
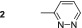	−21.59	H4‐758 ± 48 (2.4%)	H4‐21.4
		H5‐1161 ± 101 (3.6%)	H5‐23.0
		H6‐1177 ± 76 (3.7%)	H6‐29.4
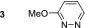	−21.63	H4‐648 ± 68 (2.0%)	H4‐28.2
		H5‐884 ± 87 (2.8%)	H5‐24.5
		H6‐759 ± 64 (2.4%)	H6‐31.0
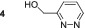	−21.54	H4‐99 ± 11 (0.3%)	H4‐12.1
		H5‐260 ± 32 (0.8%)	H5‐16.3
		H6‐273 ± 54 (0.9%)	H6‐17.6
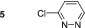	−21.52	H4‐1266 ± 105 (4.0%)	H4‐15.4
		H5‐553 ± 86 (1.7%)	H5‐20.8
		H6‐936 ± 65 (2.9%)	H6‐27.9
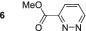	−21.42	H4‐307 ± 31 (1.0%)	H4‐22.2
		H5‐84 ± 14 (0.3%)	H5‐13.6
		H6‐252 ± 18 (0.8%)	H6‐21.7
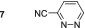	−20.68, −23.03 (*J*_HH_ = 7.3 Hz)	H4‐3 ± 1 (0.01%)	H4‐17.4
		H5‐6 ± 2 (0.02%)	H5‐25.8
		H6‐6 ± 1 (0.02%)	H6‐27.2
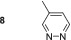	−^[a]^	H3‐613 ± 31 (1.9%)	H3‐27.2
		H5‐354 ± 19 (1.1%)	H5‐20.8
		H6‐541 ± 12 (1.7%)	H6‐24.2
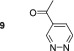	−^[a]^	H3‐564 ± 35 (1.8%)	H3‐45.4
		H5‐374 ± 48 (1.2%)	H5‐15.0
		H6‐652 ± 52 (2.0%)	H6‐17.4
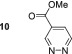	−^[a]^	H3‐862 ± 65 (2.7%)	H3‐57.4
		H5‐589 ± 71 (1.8%)	H5‐18.4
		H6‐778 ± 32 (2.4%)	H6‐18.0
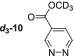	−^[a]^	H3‐1204 ± 86 (3.8%)	H3‐69.6
		H5‐754 ± 75 (2.4%)	H5‐12.2
		H6‐897 ± 101 (2.8%)	H6‐24.2

*Note*: Superscript [a] indicates a complex hydride region in the ^1^H NMR spectrum that contains a minimum of five products.

Our first step in adding complexity to the pyridazine substrate was through the introduction of a methyl group adjacent to the nitrogen atom in order to investigate steric influence on SABRE hyperpolarisation. In analogous 2‐picoline, the methyl group inhibits SABRE when using the [IrCl (COD)(IMes)] precatalyst^[^ ^22^ ^]^; however, this has been recently overcome through the use of a bidentate PHOX ligand and up to 132‐fold signal gains are reported.^[^ ^23^ ^]^ In contrast, 3‐methylpyridazine **2**, performed well under SABRE conditions and the signal gains were improved when compared with **1**. Indeed, ^1^H signal enhancements for the H4, H5 and H6 resonances were 758‐, 1161‐ and 1177‐fold, respectively. A 145‐fold signal gain was also observed for the resonance of the methyl proton. Only a single hydride containing species could be detected under both PHIP and low temperature NMR studies with a hydride resonance at *δ*_H_ −21.59. Therefore, we suggest that each of the three **2** ligands in [Ir(H)_2_(IMes)(**2**)_3_]Cl bind through the N1 atom and other potential regioisomers either do not form or are present at too low concentrations to be detected. This is likely to be due to the steric influence of the methyl group which may hinder binding through the N2 atom. 3‐Methoxypyridazine **3** also formed a single hydride containing complex and its hydride resonance shifted to *δ*_H_ −21.63 in line with the increased electron donating capacity of this ligand. After SABRE polarisation transfer under analogous conditions, a 759‐fold signal gain was observed for the H6 resonance. This is >2 times the analogous signal gain for **1**, although it is a reduction in that observed relative to **2**. Its *T*_1_ relaxation times are comparable with that of **1**.

Further complexity was introduced by utilising the hydroxymethyl functionality in **4**, and this resulted in a significantly reduced SABRE signal enhancements. Now, the maximum signal gain was 273‐fold for the H6 resonance. Although the major hydride containing species remains that of [Ir(H)_2_(IMes)(**4**)_3_]Cl (*δ*_H_ −21.54), an additional complex with broad hydride resonances at *δ*_H_ −20.68 and −30.69 also appears, which could be due to bidentate binding of **4** in the equatorial plane through its N and O donor atoms. The broadness of these resonances indicates rapid hydride loss to form H_2_, a process which would quickly consume the available *p*‐H_2_ thereby reducing the observed signal gains. Additionally, its *T*_1_ values for H4, H5 and H6 were 12.1, 16.3 and 17.6 s, respectively, and therefore shorter than those for 3‐methylpyridazine **2**.

Next, we probed the effect of introducing electron‐withdrawing substituents into the three position with substrates **5**–**7**. 3‐Chloropyridazine **5** forms a single hydride containing complex with a resonance *δ*_H_ −21.52 and performs well under SABRE. A signal gain for its H4 proton of 1266‐fold was observed, and its H5 and H6 resonances showed signal enhancements of 552‐ and 935‐fold, respectively. *T*_1_ values of 15.4, 20.8 and 27.9 s were also measured at 9.4 T. This indicates that the SABRE of monosubstituted pyridazines is tolerant to both electron‐rich and electron‐deficient ring systems. Methyl‐3‐pyridazinecarboxylate **6** did not yield as high signal enhancements as **5**. Instead, a maximum of 307‐fold signal gain was quantified for its H4 resonance. The dominant hydride containing complex gives a resonance at *δ*_H_ −21.52; however, a minor product that has hydride resonances at *δ*_H_ −20.12 and −29.42 also appears under PHIP conditions.

In contrast, 3‐pyridazinecarbonitrile **7** does not form a dihydride‐symmetric active catalyst and instead yields a product with inequivalent hydride resonances at *δ*_H_ −20.68 and −23.03 that share a *J*‐coupling of 7.3 Hz. As the binding of nitrile ligands to form an active SABRE catalyst is well established,^[^ ^24^ ^]^ we attribute these hydride resonances to the complex whereby one molecule of **7** is bound through the N1 atom and the other is bound through its nitrile group in the equatorial plane. Additionally, a minor product with hydride resonances at *δ*_H_ −20.8 and −21.9 appears under PHIP conditions, which is likely to be due to the regioisomeric binding of **7** in the axial position. In this case, poor SABRE enhancement results and a maximum 6‐fold ^1^H NMR signal enhancement was quantified at 9.4 T. The *T*_1_ values for **7** are comparable with that of pyridazine **1**.

Next, we considered the effects of 4‐substituted pyridazines **8**–**11**. 4‐Methylpyridazine **8** showed good polarisation levels of 613‐, 354‐ and 541‐fold for its H3, H5 and H6 resonances, respectively. In contrast to 3‐methylpyridazine **2**, 4‐methylpyridazine **8** yields multiple hydride‐containing complexes on reaction with [IrCl (COD)(IMes)] in the presence of H_2_. Cooling the sample to 243 K reveals >5 hydride containing complexes with resonances between *δ*_H_ –21.25 and −21.42. Because the methyl group of **8** has a reduced steric influence on the ligation to the metal centre when compared with **2**, it is now possible that the regioisomeric complexes, where binding can occur through either the substrates N1 or N2 atoms, lie much closer in energy than for the case of **2**. Statistically, this would lead to the possibility of six regioisomers of [Ir(H)_2_(IMes)(**8**)_3_]Cl being present in solution.

A complex hydride region is also present after formation of the active SABRE catalysts of 4‐acetylpyridazine **9**. Strong SABRE performance is still present and a signal gain of 564‐fold for its H3 resonance is detected. Additionally, the *T*_1_ value of the H3 resonance is increased to 45.7 s, which can be attributed to the lack of spin–spin routes to relaxation for this isolated proton. When a methyl ester is introduced into the 4‐position to give **11** an improvement in signal gain is noted. The H3, H5 and H6 resonances of **11** exhibit signal gains of 862‐, 589‐ and 778‐fold, respectively. Additionally, a 57.4 s *T*_1_ relaxation time is quantified for its H3 resonance, which is again isolated from other spins in the substrate due to the presence of the ester. Deuteration of the methyl group to form ***d***_**3**_ **–11** significantly improved its SABRE polarisation levels. Now 1204‐, 754‐ and 897‐fold signal gains were quantified for the H3, H4 and H6 resonances, respectively. The *T*_1_ relaxation values also increase, and the isolated H3 proton now has a value that is 69.6 s.

In order to increase the substrate complexity, a second substituent was added onto the pyridazine ring. First, symmetrical 3,6‐substituted pyridazines **11**–**13** were investigated followed by unsymmetrical variants **14–18** as shown in Table 2. 3,6‐*d*_2_‐Pyridazine (**3,6‐*d***_**2**_ **–1**) gave higher signal enhancements than its *protio* counterpart (**1**) with a 492‐fold per proton signal gain for the remaining H4/5 position. Additionally, the *T*_1_ relaxation time is also increased to 58.2 s from 26.6 s due to a reduction in spin–spin interactions within the substrate. 3,6‐Dimethylpyridazine (**11**) presents an increased steric challenge when compared with its mono‐substituted counterpart **2**. Although an active SABRE complex forms with a hydride resonance at *δ*_H_ −21.61, a significant reduction in polarisation transfer results. After SABRE transfer at 70 G, a 129‐fold signal gain is observed for its H4/5 proton resonance which is ca. 50% of that which was quantified for **1**. Similarly, 3,6‐dichloropyridazine **12** also forms a SABRE complex; however, its hydride resonance only becomes visible at 243 K and gives a peak at *δ*_H_ −24.45. At 298 K, a very broad hydride resonance is detected that indicates rapid ligand loss. Now, no ^1^H SABRE polarisation is detected after polarisation transfer over a range of magnetic fields (0.5–120 G). This is in contrast to the reported ^15^N signal gain of 12 000‐fold that is achieved at 8.5 T.^[^ ^21c^ ^]^ We suggest that the faster ligand dissociation is better matched for the strong ^2^ *J*_HN_ coupling between the substrate and hydride ligands in the active complex when compared with the weaker ^5^ *J*_HH_ coupling.

**TABLE 2:** Summary of SABRE hyperpolarisation and *T*_1_ relaxation times of 3,6‐disubstituted pyridazines

Substrate	Hydride resonance for [Ir(H)_2_(IMes)(sub)_3_]cl at 298 K	^1^H SABRE signal gain at 9.4 T (% polarisation)	*T*_1_ relaxation time at 298 K measured at 9.4 T
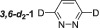	−21.50	H4/5–492 ± 35 (1.5%)	H4/5–58.2
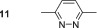	−21.61	H4/5–129 ± 14 (0.4%)	H4/5–33.4
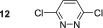	−24.45^[a]^	−	H4/5–49.4
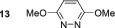	−	−	H4/5–24.1
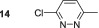	−	−	H4–26.9
			H5–18.2
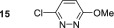	−	−	H4–30.9
			H5–29.0
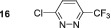	−	−	H4–22.3
			H5–26.6
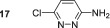	−^[b]^	H4–117 ± 21 (0.4%)	H4–16.2
		H5–31 ± (0.1%)	H5–15.2
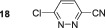	−^[b]^	H4–12 ± 3 (0.03%)	H4–26.7
		H5–3 ± 1 (0.01%)	H5–25.8

*Note*: Superscript [a] indicates chemical shift measured at 243 K. Superscript [b] indicates a complex hydride region in the ^1^H NMR spectrum that contains multiple products.

**TABLE 3:** Summary of SABRE hyperpolarisation and *T*_1_ relaxation times of 4,5‐disubstituted pyridazines

Substrate	Hydride resonance for [Ir(H)_2_(IMes)(sub)_3_]cl at 298 K	^1^H SABRE signal gain at 9.4 T (% polarisation)	*T*_1_ relaxation time at 298 K measured at 9.4 T
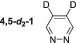	−21.59	H3/6‐1871 ± 201 (5.8%)	H3/6‐85.8
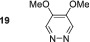	−22.12	H3/6‐998 ± 78 (3.1%)	H3/6‐7.1
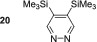	−21.00	H3/6‐128 ± 18 (0.4%)	H3/6‐8.2
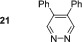	−21.15	H3/6‐823 ± (2.6%)	H3/6‐13.8
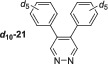	−21.15	H3/6‐1365 ± 145 (4.3%)	H3/6‐33.2
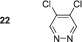	−21.03	H3/6‐878 ± 76 (2.7%)	H3/6‐84.3
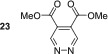	−20.89	H3/6‐1647 ± 79 (5.1%)	H3/6‐120.8
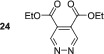	−20.84	H3/6‐1134 ± 124 (3.5%)	H3/6‐83.4
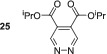	−20.89	H3/6‐465 ± 67 (1.5%)	H3/6‐68.4
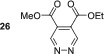	−20.87	H3/6‐1256 ± 207 (3.9%)	H3/6‐94.6
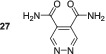	−21.20	H3/6‐421 ± 32 (1.3%)	H3/6‐39.2
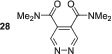	−21.20	H3/6‐325 ± 54 (1.0%)	H3/6‐12.9
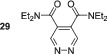	−21.24	H3/6‐237 ± 43 (0.7%)	H3/6‐5.1

No SABRE transfer is observed for 3,6‐dimethoxypyridazine (**13**) after SABRE polarisation transfer, and no evidence of any hydride containing complexes could be obtained at either low temperature or under PHIP conditions. Unsymmetrical substrates **14**–**16** also yielded no SABRE enhancements in their ^1^H NMR spectra after polarisation transfer. This indicates that the steric influence of the 3,6‐disubstituted substrates is not conducive to polarisation transfer. Finally, we were able to obtain weak SABRE enhancements for 3‐chloro‐6‐aminopyridazine **17** and 3‐chloro‐6‐cyanopyridazine **18**. In both cases, multiple hydride resonances are present in the ^1^H NMR spectrum, which reflects their multiple binding modalities. 3‐Chloro‐6‐aminopyridazine **17** showed SABRE derived signal gains of 112‐fold for the H5 signal and 61‐fold for its H4 resonance. Its ^1^H NMR spectrum reveals the presence of two dominant hydride containing complexes: first, a symmetric complex with a hydride resonance at *δ*_H_ −22.1 and second with broad resonances at *δ*_H_ −21.8 and −29.7. These complexes are formed in a 1:5 ratio and can be accounted for by binding through both the pyridazine ring nitrogen and also the aromatic amine.^[^ ^25^ ^]^ For **18**, the hydride signals are much more complex with ca. 4 hydride containing complexes being formed, which may arise due to the ability for binding through both the ring nitrogen and nitrile group. The SABRE‐derived polarisation transfer to **18** was significantly lower, and a maximum 12‐fold signal gain was quantified.

Overall, the signal enhancements for the 3,6‐disubstituted pyridazines are significantly lower than those of the monosubstituted pyridazines. This is like to be due to a combination of effects. Firstly, the occupation of the *ortho* position increases the steric demand around the metal centre, which will either inhibit the formation of the active SABRE complex or significantly reduce the lifetime of the active catalyst. Secondly, as the *ortho* sites are occupied, the SABRE process utilises the weaker ^5^ *J* coupling from the hydride to the *meta* proton which may lead to less efficacious polarisation transfer.

The investigation was then extended to 4,5‐disubstituted pyridazines which were synthesised by the well‐established^[^ ^26^ ^]^ reverse electron demand Diels–Alder reaction as detailed in the supporting information. Exposure of a methanol‐*d*_4_ solution containing [IrCl(COD)(IMes)] (5 mM), substrate **19**–**29** (20 mM) to H_2_ (3 bar) led to the formation of single hydride containing complexes in all cases with their resonances appearing between *δ*_H_ −20.89 and −22.12 for each of the substrates. We attribute these resonances to the respective product of type [Ir(H)_2_(IMes)(sub)_3_)]Cl.^[^ ^18^ ^]^ When the sample was exposed to *p*‐H_2_ (3 bar) in a ca. 70 G polarisation transfer field, significant improvements in their ^1^H NMR signal enhancements were observed when compared with the corresponding thermally polarised spectra. 4,5‐*d*_2_‐Pyridazine **4,5‐*d***_**2**_ **–1** gave a 3480‐fold per proton signal enhancement for its *ortho* resonance. Additionally, a *T*_1_ relaxation time of 85.8 s, which is longer than that of the corresponding 3,6‐*d*_2_‐pyridazine **3,6‐*d***_**2**_ **–1**, however, is shorter than that of 3,5‐*d*_2_‐pyridazine isotopologue which has been reported previously.^[^ ^19^ ^]^ Introduction of electron donating methoxy groups (**19**) into the 4,5‐positions gave signal enhancements of 998‐fold at 9.4 T for the *ortho* protons of **19**. Disappointingly, its *T*_1_ relation time significantly shortens to just 7.1 s. We propose this could be due to intramolecular dipole–dipole coupling between protons. Trimethylsilyl derivative **20** shows significantly reduced SABRE activity and just a 129‐fold ^1^H NMR signal gain was quantified at 9.4 T. Polarisation transfer was also attempted into its ^29^Si nuclei; however, no signal gain was observed after polarisation transfer at a range of fields from 0.5 G to 120 G. This is in contrast to other silyl‐derived substrates that showed SABRE polarisation transfer to ^29^Si is possible via the adjacent ^1^H nuclei at 25 G.^[^ ^27^ ^]^ The ^1^H *T*_1_ value for the *ortho* resonances was measured to be just 11.1 s. Comparatively, 4,5‐Diphenylpyridazine **21** showed good SABRE transfer, and a 823‐fold signal gain was quantified at 9.4 T. However, its *T*_1_ value was just 13.8 s. Synthesis of its selectively deuterated isotopologue yielded an improvement in both SABRE polarisation transfer and the *T*_1_ value of the remaining proton resonance. Indeed, ***d***_**10**_ **–21** gave a 1365‐fold signal gain at 9.4 T in conjunction with a relaxation time of 33.2 s were quantified.

When electronegative atoms are placed in the 4,5‐positions, good SABRE enhancement is still evident. Thus, when 4,5‐dichloropyridazine **22** reduced the signal gain was 878‐fold and its *T*_1_ value increased to 84.3 s. Other electron‐withdrawing groups performed better with dimethyl ester **23** yielding a 1647‐fold signal gain for its *ortho* resonance, and the relaxation time was to 120.1 s. This is equivalent to 5.1% polarisation at 9.4 T.

As this diester derivative showed strong polarisation transfer and long magnetic lifetimes, we wished to further probe the effect of the ester moiety on the SABRE effect. Therefore, diethyl ester **24** was synthesised and subject to analogous SABRE testing. After interrogation at 9.4 T a 1134‐fold signal enhancement resulted. This is a reduction when compared with dimethyl ester **23** and a reduction in *T*_1_ value from 120.8 to 83.4 s is also noted. Introduction of isopropyl esters (**25**) further reduces both the SABRE signal gains and the *T*_1_ values. These *T*_1_ effects can be quantified by working out an enhancement value at the point of creation of the hyperpolarised state. Assuming this *T*_1_ value remains constant for 5 s during the moving into the measurement field where the polarisation level is quantified, we estimate that initial polarisation levels of ca. 1700‐, 1250‐ and 500‐fold for **23**, **24** and **25** respectively result. This indicates that there is a steric effect taking place during the SABRE process even in the remote 4,5‐positions. Breaking the symmetry of the molecule through the use of one methyl ester and one ethyl ester in **26** returns the substrate to high average SABRE enhancements of 1256. The individual enhancements for H3 and H6 could not be determined due to peak overlap. The average *T*_1_ on the remaining proton sites is now 94.6 s.

The introduction of amide groups into the 4,5‐positions reduces the SABRE performance of the pyridazine molecules **27–29** when compared with dimethyl ester **23**. Additionally, the *T*_1_ values for the amide derivatives are all significantly smaller than the ester counterparts, which may reduce the detected polarisation level. A similar *T*_1_ analysis reveals that at the point of creation, the polarisation levels for **27**, **28** and **29** would be ca. 500‐, 500‐ and 550‐fold, respectively. These values are all comparable with one another and yet three times smaller than those calculated for dimethyl ester **23**. Such an effect is likely to be due to an increase in the ligand exchange rate relative to that of the ester derivate and away from that which has been predicted to be optimal by Barskiy.^[^ ^28^ ^]^ Given the previously reported studies on SABRE as a function of electronic and steric NHC impact, such behaviour is not unexpected.^[^ ^15^ ^]^ Analysis of the reported values for nicotinamide and methyl nicotinate reveals a similar 3‐fold reduction in signal enhancement where electronic effects are clearly dominant.^[^ ^19^ ^]^

**FIGURE 1:**
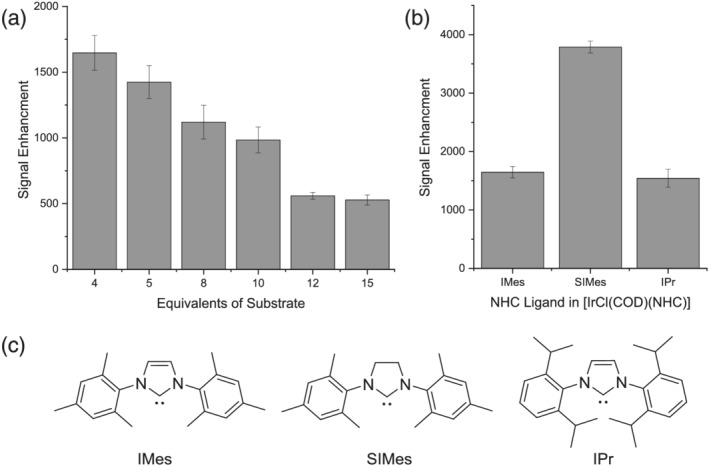
Optimisation of SABRE hyperpolarisation of **23**. (a) Effect of increasing the equivalents of substrate on the SABRE hyperpolarisation of **23**. (b) Effect of changing the *N*‐heterocyclic carbene (NHC) ligand in the precatalyst of type [IrCl (COD)(NHC)] on the SABRE hyperpolarisation of **23**. (c) Structures of NHC ligands used in the study

With the success of SABRE hyperpolarisation of dimethyl ester **23**, we began to explore methods to further optimise its hyperpolarisation and explore the effects of other carbonyl containing substrates. First, we investigated the effect of increasing the concentration of **23** on the resulting SABRE signal gains (Figure 1). We found that four equivalents of **23** was optimal and increasing beyond this catalyst loading led to a reduction in polarisation efficacy. This is consistent with the effects of diluting the finite available polarisation from *p*‐H_2_ through an increased number of substrate spins.

The nature of the *N*‐heterocyclic carbene (NHC) in the SABRE precatalyst can have a profound effect on the signal enhancement.^[^ ^15^, ^29^ ^]^ Therefore, we screened a series of precatalysts of type [IrCl(COD)(NHC)], which have been shown to modulate the rate of ligand loss through increased steric effects.^[^ ^29b^ ^]^ When using [IrCl (COD)(SIMes)] as the precatalyst an increased signal enhancement of 3788 ± 132‐fold was observed for **23**, which is over two times larger than that seen with [IrCl(COD)(IMes)]. However, further utilisation of the larger IPr ligand led to a reduction in signal enhancement to 1542 ± 154‐fold. Therefore, we can conclude that the optimal conditions for the polarisation of **23** are [IrCl(COD)(SIMes)] (5 mM), **23** (20 mM) in methanol‐*d*_4_, and these conditions would be applied to further studies on the 4,5‐dicarbonyl pyridazines.

**FIGURE 2:**
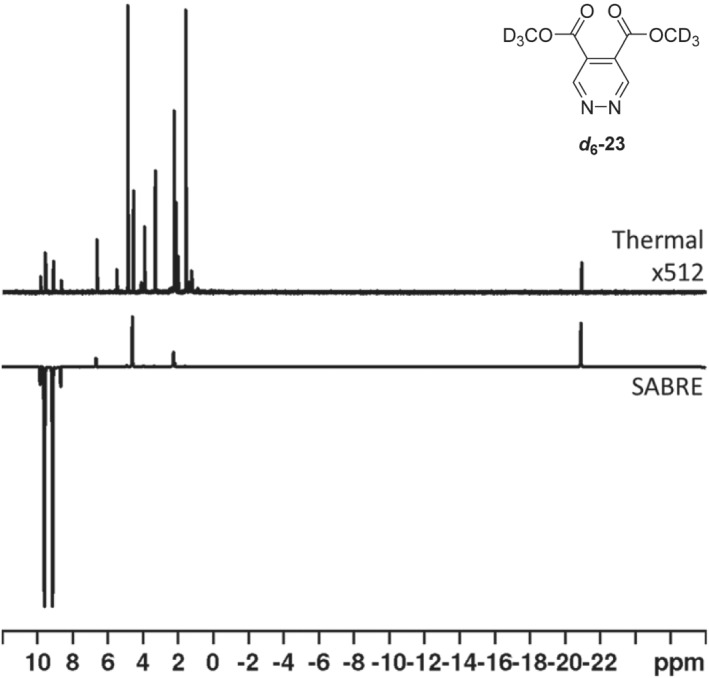
Single scan ^1^H NMR spectra of ***d***_**6**_ **–23** under (top) thermally polarised conditions that has been expanded 512× in the vertical direction and (bottom) after SABRE polarisation transfer at 70 G using [IrCl (COD)(IMes)] (5 mM), ***d***_**6**_ **–23** (20 mM) in methanol‐*d*_4_

We began with the synthesis of its isotopologue, ***d***_**6**_ **–23**, with a view to extending its relaxation lifetime and increasing its SABRE efficacy. After subjection of ***d***_**6**_ **–23** to the optimised SABRE conditions ([IrCl(COD)(SIMes)] (5 mM), ***d***_**6**_ **–23** (20 mM) in methanol‐*d*_4_), a 9177‐fold per proton signal gain was observed for its *ortho* resonances at 9.4 T which is equivalent to 28.7% polarisation (Figure 2). Additionally, a *T*_1_ relaxation time of 138.4 s was recorded.

## 3 CONCLUSIONS

In summary, we present a general method for the SABRE hyperpolarisation of over 30 substituted pyridazines and signal enhancements of up to 9177‐fold at 9.4 T. This is equivalent to 28.7% polarisation and shows the potential of this method to dramatically improve signal strength in magnetic resonance techniques. Monosubstituted pyridazines perform well and yield good signal enhancements. The steric effect of adding groups into the 3‐position is minimised through the substrates' ability to bind through the N1 atom. The method is able to tolerate not only electron‐rich pyridazines such as 3‐methoxypyridazine **3** but also electron poor substrates such as 3‐chloropyridazine **4**. Similarly, 4‐substituted pyridazines perform equally well. Interestingly, when the substrate contains the possibility for multiple binding modalities, through additional groups such as alcohols or nitriles, the active catalyst differs from that of the typical symmetric [Ir(H)_2_(IMes)(sub)_3_]Cl. For example, 3‐cyanopyridazine **7** formed a catalyst where one of the equatorial ligands binds through the N1 atom and the second ligates via the nitrile moiety. This results in lower SABRE signal enhancements when compared with pyridazine **1**.

The hyperpolarisation of pyridazines becomes more sensitive when increased steric bulk is placed into the 3‐ and 6‐positions on the aromatic ring. Indeed, when 3,6‐dimethylpyridazine **11** is utilised, a 129‐fold signal gain is observed which is a significant reduction when compared with 3‐methylpyridazine **2** (up to 1177‐fold signal gain). Increasing the steric bulk further leads to no signal enhancements being observed at 298 K, and no evidence of any hydride containing species could be detected. This suggests that the NHC‐derived catalysts commonly utilised for SABRE cannot form under the conditions used here, and therefore, the exploration of other catalysts could be fruitful.^[^ ^23^, ^30^ ^]^

In contrast, 4,5‐disubstituted pyridazines all form the expected symmetric [Ir(H)_2_(IMes)(sub)_3_]Cl catalyst and thus undergo efficacious SABRE. The electronic effect of changing the functionality does not appear to significantly impact the resulting signal enhancements after polarisation transfer. For example, 4,5‐dimethoxypyridazine **19** shows an excellent per proton signal gain for its H3/H6 resonance of 998‐fold, whereas for 4,5‐dichloropyridazine, **22** yielded a signal gain of 878‐fold. The best performing substrate was dimethylester **23** which gave a signal gain of 1647‐fold in conjunction with a *T*_1_ relaxation time of over 2 min at 9.4 T.

Finally, we exemplify the possibility to further increase the SABRE transfer to substituted pyridazines by optimisation of the polarisation level of **23**. We found that changing the precatalyst to [IrCl(COD)(SIMes)] a higher signal gain could be achieved and deuteration of the methyl esters to give ***d***_**6**_ **–23** ultimately yielded a polarisation level of >28%. Deuteration of the methyl esters also extended the *T*_1_ relaxation time to 138 s. The data reported here will allow for the development of methods to hyperpolarise and detect a range of pyridazine containing pharmaceutical and agrochemicals such as the antidepressant minaprine or anticonvulent SR41378.^[^ ^3^ ^]^ Next stages will see us consider the effect of fused ring systems and seek to overcome the challenges posed by the steric influence of 3,6‐disubstituted systems.

4

### PEER REVIEW

The peer review history for this article is available at https://publons.com/publon/10.1002/mrc.5152.

## Supporting information


**Figure S1.** NMR spectra of [IrCl (COD)(IMes)] (5 mM), 3‐methylpyridazine **2** (20 mM) in methanol‐*d*_4_ under thermally polarised conditions (top) and after SABRE polarisation transfer under *p*‐H_2_ (3 bar) at 70 G (bottom).
**Figure S2.** NMR spectra of [IrCl (COD)(IMes)] (5 mM), 3‐cyanopyridazine **7** (20 mM) in methanol‐*d*_4_ under thermally polarised conditions (top) and after SABRE polarisation transfer under *p*‐H_2_ (3 bar) at 70 G (bottom).
**Figure S3.** NMR spectra of [IrCl (COD)(IMes)] (5 mM), 4‐methylpyridazine **8** (20 mM) in methanol‐*d*_4_ under thermally polarised conditions (top) and after SABRE polarisation transfer under *p*‐H_2_ (3 bar) at 70 G (bottom).
**Figure S4.** NMR spectra of [IrCl (COD)(IMes)] (5 mM), 4,5‐dimethyl pyridazine‐4,5‐dicarboxylate **23** (20 mM) in methanol‐*d*_4_ under thermally polarised conditions (top) and after SABRE polarisation transfer under *p*‐H_2_ (3 bar) at 70 G (bottom).
**Figure S5.** NMR spectra of [IrCl (COD)(IMes)] (5 mM), 4,5‐bis (methyl‐d_3_) pyridazine‐4,5‐dicarboxylate ***d***_**6**_ **–23** (20 mM) in methanol‐*d*_4_ under thermally polarised conditions (top) and after SABRE polarisation transfer under *p*‐H_2_ (3 bar) at 70 G (bottom).
**Figure S6.** NMR spectra of [IrCl (COD)(IMes)] (5 mM), *N*,*N*,*N*′,*N*′‐Tetraethyl pyridazine 4,5‐dicarboxamide **28** (20 mM) in methanol‐*d*_4_ under thermally polarised conditions (top) and after SABRE polarisation transfer under *p*‐H_2_ (3 bar) at 70 G (bottom).
**Figure S7.** ^1^H NMR spectrum of 4‐*d*_3_‐methyl pyridazine 4‐carboxylate ***d***_**3**_ **–10**.
**Figure S8.** ^13^C{^1^H} NMR spectrum of 4‐*d*_3_‐methyl pyridazine 4‐carboxylate ***d***_**3**_ **–10**.
**Figure S9.** ^1^H NMR spectrum of 4,5‐bis (trimethylsilyl) pyridazine **20**.
**Figure S10.** ^13^C{^1^H} NMR spectrum of 4,5‐bis (trimethylsilyl) pyridazine **20**.
**Figure S11.** ^1^H NMR spectrum of 4,5‐diphenyl pyridazine **21**.
**Figure S12.** ^13^C{^1^H} NMR spectrum of 4,5‐diphenyl pyridazine **21**.
**Figure S13.** ^1^H NMR spectrum of 4,5‐diphenyl pyridazine *d*_10_–**21**.
**Figure S14.** ^13^C{^1^H} NMR spectrum of 4,5‐diphenyl pyridazine *d*_10_–**21**.
**Figure S15.** ^1^H NMR spectrum of 4,5‐dimethyl pyridazine 4,5‐dicarboxylate **23**.
**Figure S16.** ^13^C{^1^H} NMR spectrum of 4,5‐dimethyl pyridazine 4,5‐dicarboxylate **23**.
**Figure S17.** ^1^H NMR spectrum of 4,5‐bis (*d*_3_‐methyl) pyridazine 4,5‐dicarboxylate *d*_6_–**23**.
**Figure S18.** ^13^C{^1^H} NMR spectrum of 4,5‐bis (*d*_3_‐methyl) pyridazine 4,5‐dicarboxylate *d*_6_–**23**.
**Figure S19.** ^1^H NMR spectrum of 4‐methyl 5‐ethyl pyridazine 4,5‐dicarboxylate **26**.
**Figure S20.** ^13^C{^1^H} NMR spectrum of 4‐methyl 5‐ethyl pyridazine 4,5‐dicarboxylate **26**.
