# Supplementary material for: Steric and electronic effects on the 1H hyperpolarisation of substituted pyridazines by signal amplification by reversible exchange
Source: Magn Reson Chem. 2021 Apr 5;59(12):1187–98. doi: 10.1002/mrc.5152 (PMC8650576; doi:10.1002/mrc.5152)
Supplement: Supplementary file 1 — Figure S1. NMR spectra of [IrCl (COD)(IMes)] (5 mM), 3‐methylpyridazine 2 (20 mM) in methanol‐d 4 under thermally polarised conditions (top) and after SABRE polarisation transfer under p‐H2 (3 bar) at 70 G (bottom). Figure S2. NMR spectra of [IrCl (COD)(IMes)] (5 mM), 3‐cyanopyridazine 7 (20 mM) in methanol‐d 4 under thermally polarised conditions (top) and after SABRE polarisation transfer under p‐H2 (3 bar) at 70 G (bottom). Figure S3. NMR spectra of [IrCl (COD)(IMes)] (5 mM), 4‐methylpyridazine 8 (20 mM) in methanol‐d 4 under thermally polarised conditions (top) and after SABRE polarisation transfer under p‐H2 (3 bar) at 70 G (bottom). Figure S4. NMR spectra of [IrCl (COD)(IMes)] (5 mM), 4,5‐dimethyl pyridazine‐4,5‐dicarboxylate 23 (20 mM) in methanol‐d 4 under thermally polarised conditions (top) and after SABRE polarisation transfer under p‐H2 (3 bar) at 70 G (bottom). Figure S5. NMR spectra of [IrCl (COD)(IMes)] (5 mM), 4,5‐bis (methyl‐d3) pyridazine‐4,5‐dicarboxylate d 6 –23 (20 mM) in methanol‐d 4 under thermally polarised conditions (top) and after SABRE polarisation transfer under p‐H2 (3 bar) at 70 G (bottom). Figure S6. NMR spectra of [IrCl (COD)(IMes)] (5 mM), N,N,N′,N′‐Tetraethyl pyridazine 4,5‐dicarboxamide 28 (20 mM) in methanol‐d 4 under thermally polarised conditions (top) and after SABRE polarisation transfer under p‐H2 (3 bar) at 70 G (bottom). Figure S7. 1H NMR spectrum of 4‐d 3‐methyl pyridazine 4‐carboxylate d 3 –10. Figure S8. 13C{1H} NMR spectrum of 4‐d 3‐methyl pyridazine 4‐carboxylate d 3 –10. Figure S9. 1H NMR spectrum of 4,5‐bis (trimethylsilyl) pyridazine 20. Figure S10. 13C{1H} NMR spectrum of 4,5‐bis (trimethylsilyl) pyridazine 20. Figure S11. 1H NMR spectrum of 4,5‐diphenyl pyridazine 21. Figure S12. 13C{1H} NMR spectrum of 4,5‐diphenyl pyridazine 21. Figure S13. 1H NMR spectrum of 4,5‐diphenyl pyridazine d 10–21. Figure S14. 13C{1H} NMR spectrum of 4,5‐diphenyl pyridazine d 10–21. Figure S15. 1H NMR spectrum of 4,5‐dimethyl pyridazi [file MRC-59-1187-s001.docx]

Supporting Information

Steric and Electronic Effects on the ^1^H Hyperpolarisation of Substituted Pyridazines by Signal Amplification by Reversible Exchange

Peter J. Rayner, Michael J. Burns, Elizabeth J. Fear and Simon B. Duckett

**1 NMR polarisation transfer experiment data**

**1.1** **SABRE polarisation transfer method**

The polarisation transfer experiments that are reported were conducted in 5 mm NMR tubes that were equipped with a J. Young’s tap. Samples for these polarisation transfer experiments were based on a 5 mM solution of [IrCl(COD)(NHC)], substrate (20 mM) in methanol-*d*_4_ unless otherwise stated. The samples were degassed by two freeze-pump-thaw cycles prior to the introduction of *para*hydrogen at a pressure of 3 bar. *Para*-hydrogen (*p*-H_2_) was produced by passing hydrogen gas over a spin-exchange catalyst (Fe_2_O_3_) at 28 K and used for all hyperpolarization experiments. This method produces constant *p*-H_2_ with ca. 98% purity.

The shake & drop method was employed for recording hyperpolarized SABRE NMR spectra.[^1^](#_ENREF_1) Once filled with *p*-H_2_, samples were shaken vigorously for 10 s in the specified fringe field of an NMR spectrometer before being rapidly transported into the magnet for subsequent interrogation by NMR spectroscopy.

**1.2 Polarization factors**

^1^H signal enhancements were calculated according to equation 1 where, E = enhancement level, SI(pol) = signal of polarized sample, SI(unpol) = signal of unpolarized (reference) sample.

$$E=\frac{SI(pol)}{SI(unpol)} (1)$$

Experimentally, both spectra were recorded on the same sample using identical acquisition parameters, including the receiver gain. The raw integrals of the relevant resonances in the polarized and unpolarised spectra were then used to determine the enhancement levels. The quoted values reflect the signal strength gain (fold) per proton nucleus in the specified group. The reference sample was allowed to equilibrate within the NMR spectrometer for 1-2 minutes prior to acquisition.

**1.3 NMR Spectrometer**

Spectra were typically acquired on a 400 MHz Bruker, Avance III console using a 5 mm BBI probe which was tuned to ^1^H. Resonances are referenced relative to the residual proton signal of the indicated deuterated solvent.

**2. Representative Examples of Hyperpolarised NMR spectra**

**
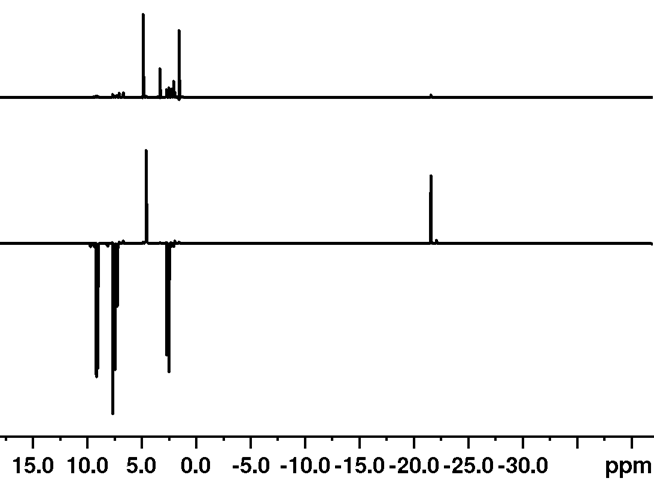
**

Figure S1: NMR spectra of [IrCl(COD)(IMes)] (5 mM), 3-methylpyridazine **2** (20 mM) in methanol-*d*_4_ under thermally polarised conditions (top) and after SABRE polarisation transfer under *p*-H_2_ (3 bar) at 70 G (bottom).

**
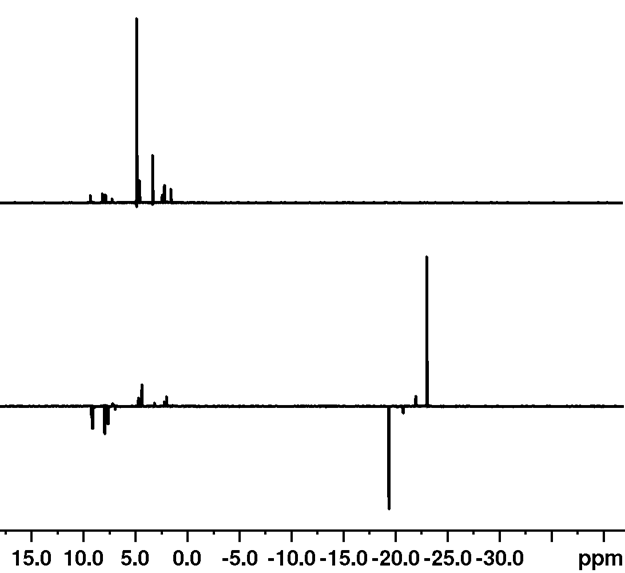
**

Figure S2: NMR spectra of [IrCl(COD)(IMes)] (5 mM), 3-cyanopyridazine **7** (20 mM) in methanol-*d*_4_ under thermally polarised conditions (top) and after SABRE polarisation transfer under *p*-H_2_ (3 bar) at 70 G (bottom).

**
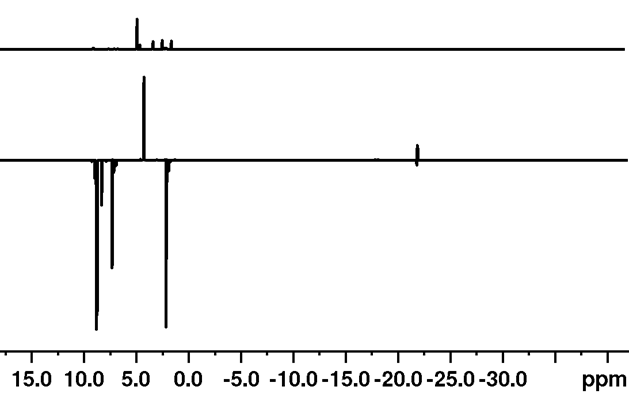
**

Figure S3: NMR spectra of [IrCl(COD)(IMes)] (5 mM), 4-methylpyridazine **8** (20 mM) in methanol-*d*_4_ under thermally polarised conditions (top) and after SABRE polarisation transfer under *p*-H_2_ (3 bar) at 70 G (bottom).

**
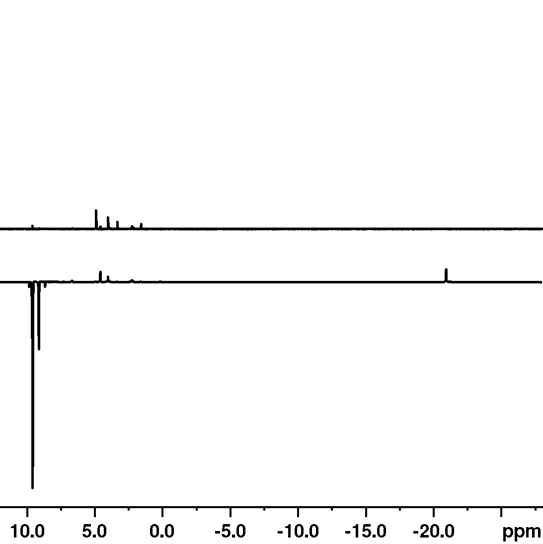
**

Figure S4: NMR spectra of [IrCl(COD)(IMes)] (5 mM), 4,5-dimethyl pyridazine-4,5-dicarboxylate **23** (20 mM) in methanol-*d*_4_ under thermally polarised conditions (top) and after SABRE polarisation transfer under *p*-H_2_ (3 bar) at 70 G (bottom).

**
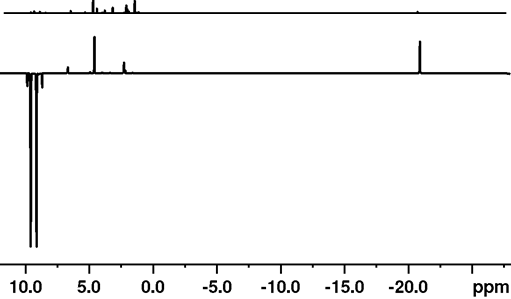
**

Figure S5: NMR spectra of [IrCl(COD)(IMes)] (5 mM), 4,5-bis(methyl-d_3_) pyridazine-4,5-dicarboxylate ***d*_6_-23** (20 mM) in methanol-*d*_4_ under thermally polarised conditions (top) and after SABRE polarisation transfer under *p*-H_2_ (3 bar) at 70 G (bottom).

**
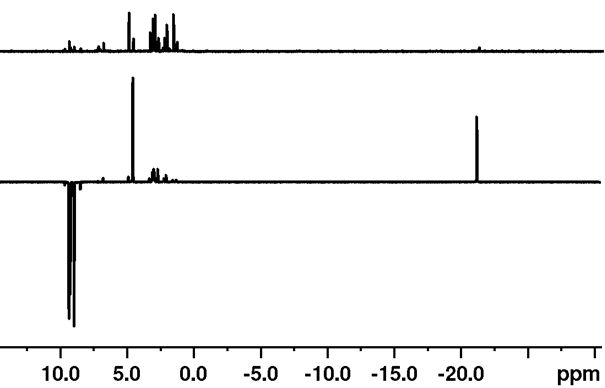
**

Figure S6: NMR spectra of [IrCl(COD)(IMes)] (5 mM), *N*,*N*,*N*’,*N*’-Tetraethyl pyridazine 4,5-dicarboxamide **28** (20 mM) in methanol-*d*_4_ under thermally polarised conditions (top) and after SABRE polarisation transfer under *p*-H_2_ (3 bar) at 70 G (bottom).

**3. Synthetic Details**

**3.1 General**

Water is distilled water. Brine refers to a saturated aqueous solution of NaCl. THF was freshly distilled from sodium and benzophenone ketyl or dried using a Grubbs solvent purification system. Petrol refers to the fraction of petroleum ether boiling in the range 40-60 °C. All reactions were carried out under O_2_-free Ar or N_2_ using oven-dried and/or flame-dried glassware.

Flash column chromatography was carried out using Fluka Chemie GmbH silica (220-440 mesh). Reverse-phase flash column chromatography was carried out using a Biotage Isolera with a SNAP-C_18_-12g cartridge eluting with H_2_O-MeCN containing 0.1% NH_4_OH. Thin layer chromatography was carried out using Merck F_254_ aluminium-backed silica plates. ^1^H (400 MHz) and ^13^C (100.6 MHz) NMR spectra were recorded on a Bruker-400 instrument with an internal deuterium lock. Chemical shifts are quoted as parts per million and referenced to CHCl_3_ (δ_H_ 7.27), (CH_3_)_2_SO (δ_H_ 2.54), CDCl_3_ (δ_C_ 77.0) or (CD_3_)_2_SO (δ_C_ 40.45). ^13^C NMR spectra were recorded with broadband proton decoupling. ^13^C NMR spectra were assigned using DEPT experiments. Coupling constants (*J*) are quoted in Hertz. Electrospray high and low resolution mass spectra were recorded on a Bruker Daltronics microOTOF spectrometer.

All compounds were purchased from Sigma-Aldrich, Fluorochem or Alfa-Aesar and used as supplied unless otherwise stated. The following compounds were synthesised according to literature procedures; 1,2,4,5-tetrazine,[^2^](#_ENREF_2) [IrCl(COD)(IMes)],[^3^](#_ENREF_3) 4,5-dimethoxypyridazine **19**,[^4^](#_ENREF_4) and pyridazine-4,5-dicarboxamide **27**.[^5^](#_ENREF_5)

**3.2 Experimental Procedures**

**4-Methyl-*d*_3_ pyridazine 4-carboxylate *d*_3_-10**

Oxalyl chloride (0.81 mL, 9.67 mmol, 1.2 eq.) was added to a stirred suspension of pyridazine 4-carboxylic acid (1.00 g, 8.06 mmol, 1.0 eq.) in CH_2_Cl_2_ (25 mL) at rt under N_2_. Then, DMF (two drops) were added and the solution was stirred at rt for 1 h. The reaction was concentrated under reduced pressure. The residue was dissolved in CH_2_Cl_2_ (25 mL) and trimethylamine (1.37 mL, 10.48 mmol, 1.3 eq.) and CD_3_OD (2 mL) were added sequentially. The reaction was concentrated under reduced pressure and EtOAc (20 mL) and sat. NaHCO_3(aq)_ (20 mL) were added and the two layers separated. The aqueous layer was extracted with EtOAc (2 x 20 mL) and the combined organic layers were dried (MgSO_4_) and concentrated under reduced pressure to give the crude product. Purification by flash column chromatography on silica with 9:1 CH_2_Cl_2_-EtOAc as eluent gave ***d*_3_-10** (988 mg, 87%), ^1^H NMR (400 MHz, CD_3_CD_2_OD) 9.65 (t, *J* = 1.65 Hz, 1H), 9.42 (dd, *J* = 5.2, 1.4 Hz, 4H), 7.98 (dd, *J* = 5.3, 1.8 Hz, 6H); ^13^C NMR (100.6 MHz, CDCl_3_) *δ* 164.2, 152.1, 149.7, 127.7, 125.5, 52.5 (*sept*. , *J* = 21.1 Hz); MS (ESI) *m/z* 164 [(M + Na)^+^, 100 ], 142 [(M + H)^+^, 30];HRMS *m/z* calcd for C_6_H_3_D_3_N_2_O_2_ (M + Na)^+^ 164.0510, found 164.0510 (−0.4 ppm error).

**4,5-Bis(trimethylsilyl) pyridazine 20**

Bis(trimethylsilyl) acetylene (176 mg, 1.03 mmol, 1.03 eq.) was added to a stirred solution of 1,2,4,5-tetrazine (82 mg, 1.00 mmol, 1.0 eq.) in MeCN (2.5 mL) at rt under N_2_(g). The resulting solution was heated to 80 °C for 16 h. The reaction was cooled to rt and concentrated under reduced pressure to give the crude product. Purification flash column chromatography on silica with 8:2 pentane-EtOAc gave **20** (196 mg, 88%), ^1^H NMR (500 MHz, CD_3_OD) 9.19 (s, 2H), 0.48 (s, 18H); ^13^C NMR (126 MHz, CD_3_OD) *δ* 153.8, 147.0, −0.77; MS (ESI) *m/z* 247 [(M + Na)^+^, 50 ], 224 [(M + H)^+^, 100]; HRMS *m/z* calcd for C_10_H_20_N_2_Si_2_ (M + Na)^+^ 247.1057, found 247.1050 (+3.3 ppm error); Spectroscopic data consistent with those reported in the literature.[^2^](#_ENREF_2)

**4,5-Diphenyl pyridazine 21**

Diphenyl acetylene (183 mg, 1.03 mmol, 1.03 eq.) and 1,2,4,5-tetrazine (82 mg, 1.00 mmol, 1.0 eq.) in toluene (2.5 mL) was heated under microwave irradiation at 180 °C for 2 h. The reaction mixture was concentrated under reduced pressure to give the crude product. Purification by flash column chromatography on silica with of 7:3 petrol-EtOAc gave **21** (150 mg, 65%), *R*_F_ (7:3 petrol-EtOAc) 0.2; ^1^H NMR (500 MHz, CD_3_OD) 9.23 (s, 2H), 7.43-7.36 (m, 6H), 7.32-7.29 (m, 4H); ^13^C NMR (126 MHz, CD_3_OD) *δ* 151.7, 138.5, 134.5, 129.2, 128.8, 128.5; MS (ESI) *m/z* 255[(M + Na)^+^, 100 ], 233 [(M + H)^+^, 80]; HRMS *m/z* calcd for C_16_H_12_N_2_ (M + Na)^+^ 255.0893, found 255.0893 (−0.4 ppm error).

**4,5-Bis(*d*_5_-phenyl) pyridazine *d*_10_-21**

*d*_10_-Diphenyl acetylene (194 mg, 1.03 mmol, 1.03 eq.) and 1,2,4,5-tetrazine (82 mg, 1.00 mmol, 1.0 eq.) in toluene (2.5 mL) was heated under microwave irradiation at 180 °C for 2 h. The reaction mixture was concentrated under reduced pressure to give the crude product. Purification by flash column chromatography on silica with of 7:3 petrol-EtOAc gave ***d*_10_-21** (178 mg, 74%), *R*_F_ (7:3 petrol-EtOAc) 0.2; ^1^H NMR (500 MHz, CD_3_OD) 9.24 (s, 2H); ^13^C NMR (126 MHz, CD_3_OD) *δ* 151.7, 138.5, 134.3, 128.8 (t, *J* = 24.4 Hz), 128.3 (t, *J* = 25.0 Hz), 128.1 (t, *J* = 25.0 Hz); MS (ESI) *m/z* 265 [(M + Na)^+^, 95 ], 243 [(M + H)^+^, 100]; HRMS *m/z* calcd for C_16_H_2_D_10_N_2_ (M + Na)^+^ 265.1520, found 265.1521 (−0.1 ppm error).

**4,5-Dimethyl pyridazine-4,5-dicarboxylate 23**

Me_3_SiCHN_2_ (1.1 mL of 2.0 M in hexanes, 2.20 mmol, 2.2 eq.) was added dropwise to a stirred suspension of 4,5-pyridazine dicarboxylic acid (168 mg, 1.0 mmol, 1.0 eq.) in THF (5 mL) and MeOH (1 mL) at rt under N_2_(g). The resulting solution was stirred at rt for 2 h. Then, MeOH (5 mL) was added and the reaction was concentrated under reduced pressure to give the crude product. Purification by flash column chromatography on silica with 9:1 CH_2_Cl_2_-EtOAc gave **23** (161 mg, 82%), ^1^H NMR (400 MHz, CDCl_3_) 9.38 (s, 2H), 3.86 (s, 6H); ^13^C NMR (100.6 MHz, CDCl_3_) *δ* 164.2, 149.1, 127.8, 53.6; MS (ESI) *m/z* 219 [(M + Na)^+^, 100 ], 197 [(M + H)^+^, 65]; HRMS *m/z* calcd for C_8_H_8_N_2_O_4_ (M + Na)^+^ 219.0376, found 219.0377 (−0.2 ppm error); Spectroscopic data consistent with those reported in the literature.[^5^](#_ENREF_5)

**4,5-Bis(methyl-*d*_3_) pyridazine 4,5-dicarboxylate *d*_6_-23**

Me_3_SiCHN_2_ (2.5 mL of 2.0 M in hexane, 5.0 mmol, 5 eq.) was stirred in MeOD (10 mL) at rt for 5 h under N_2_(g). This solution was then added to a stirred suspension of 4,5-pyridazine dicarboxylic acid (168 mg, 1.0 mmol, 1.0 eq.) in MeOD (5 mL). The resulting solution was stirred at rt for 3 h. Then, the reaction was concentrated under reduced pressure to give the crude product. Purification by flash column chromatography on silica 9:1 CH_2_Cl_2_-EtOAc gave ***d*_6_-23** (134 mg, 66%), ^1^H NMR (400 MHz, CDCl_3_) 9.44 (s, 1H); ^13^C NMR (100.6 MHz, CDCl_3_) *δ* 164.2, 149.1, 127.7, 52.9 (sept., *J* = 22.5 Hz); MS (ESI) *m/z* 225 [(M + Na)^+^, 100 ], 203 [(M + H)^+^, 40]; HRMS *m/z* calcd for C_8_H_2_D_6_N_2_O_4_ (M + Na)^+^ 225.0753, found 25.0761 (−3.3 ppm error).

**4,5-Diethyl pyridazine 4,5-dicarboxylate 24**

Et_3_N (0.5 mL) was added to a stirred suspension of pyridazine-4,5-dicarboxylic acid (200 mg, 1.2 mmol, 1.0 eq.) and EtOH (0.5 mL) in THF (10 mL) at rt. The resulting solution was stirred at rt for 15 min and then T3P^®^ (2.0 mL of a 50% solution in THF) was added dropwise. The reaction was stirred at rt for 16 h. Then, a saturated solution of NaHCO_3_ (15 mL) and CH_2_Cl_2_ (15 mL) were added and the two layers separated. The aqueous layer was extracted with CH_2_Cl_2_ (2 x 15 mL) and the combined organic layers were dried (MgSO_4_) and concentrated under reduced pressure to give the crude product. Purification by flash column chromatography on silica with 9:1 CH_2_Cl_2_-EtOAc as eluent gave **24** (121 mg, 40%), ^1^H NMR (400 MHz, CD_3_CD_2_OD) 9.56 (s, 2H), 4.47 (q, *J* = 8.0 Hz, 4H), 1.42 (t, *J* = 8.0 Hz, 6H); ^13^C NMR (100.6 MHz, CDCl_3_) *δ* 163.6, 149.3, 128.5, 62.8, 13.3; MS (ESI) *m/z* 247 [(M + Na)^+^, 100 ], 225 [(M + H)^+^, 55]; HRMS *m/z* calcd for C_10_H_12_N_2_O_4_ (M + Na)^+^ 247.0695, found 247.0697 (+2.1 ppm error).

**4,5-Diisopropyl pyridazine 4,5-dicarboxylate 25**

Et_3_N (0.5 mL) was added to a stirred suspension of pyridazine-4,5-dicarboxylic acid (200 mg, 1.2 mmol, 1.0 eq.) and ^i^PrOH (0.5 mL) in THF (10 mL) at rt. The resulting solution was stirred at rt for 15 min and then T3P^®^ (2.0 mL of a 50% solution in THF) was added dropwise. The reaction was stirred at rt for 16 h. Then, a saturated solution of NaHCO_3_ (15 mL) and CH_2_Cl_2_ (15 mL) were added and the two layers separated. The aqueous layer was extracted with CH_2_Cl_2_ (2 x 15 mL) and the combined organic layers were dried (MgSO_4_) and concentrated under reduced pressure to give the crude product. Purification by flash column chromatography on silica with 9:1 CH_2_Cl_2_-EtOAc as eluent gave **25** (142 mg, 47%), ^1^H NMR (400 MHz, CD_3_CD_2_OD) 9.55 (s, 2H), 1.82 (sept., *J* = 7.5 Hz, 2H), 1.04 (t, *J* = 7.5 Hz, 12H); MS (ESI) *m/z* 275 [(M + Na)^+^, 100 ], 253 [(M + H)^+^, 25]; HRMS *m/z* calcd for C_12_H_16_N_2_O_4_ (M + Na)^+^ 275.1008, found 275.1003 (+4.7 ppm error).

**4-Methyl 5-ethyl pyridazine 4,5-dicarboxylate 26**

30% H_2_O_2_ (2.7 g, 15.0 mmol, 3.0 eq.) was added to ethyl pyruvate (2.61 g, 22.5 mol, 4.5 eq.) at −10 °C. This solution was then added to methyl pyridazine-4-carboxylate (691 mg, 5.0 mmol, 1.0 eq.) at 0 °C followed by sequential addition of conc. H_2_SO_4_ (1.5 g), water (4.0 mL), FeSO_4_.7H_2_O (4.15 g, 15.0 mmol, 3.0 eq.) and CH_2_Cl_2_ (20 mL). The resulting solution was stirred at 0 °C for 15 min. The reaction mixture was poured into ice-water and extracted with CH_2_Cl_2_ (3 x 25 mL). The combined organic layers were dried (MgSO_4_) and concentrated under reduced pressure to give the crude product. Purification by flash column chromatography on silica with 9:1 CH_2_Cl_2_-EtOAc gave **26** (808 mg, 77%), ^1^H NMR (400 MHz, CDCl_3_) 9.45 (d, *J* = 1.1 Hz, 1H), 9.43 (d, *J* = 1.1 Hz, 1H), 4.40 (q, *J* = 7.0 Hz, 2H), 3.93 (s, 3H), 1.34 (t, *J* = 7.0 Hz, 3H); ^13^C NMR (100.6 MHz, CDCl_3_) *δ* 164.3, 163.7, 149.2, 149.1, 127.9, 127.7, 63.1, 53.5, 14.0; MS (ESI) *m/z* 233 [(M + Na)^+^, 100 ], 211 [(M + H)^+^, 50]; HRMS *m/z* calcd for C_9_H_11_N_2_O_4_ (M + Na)^+^ 233.0533, found 233.0533 (−0.5 ppm error).

***N*,*N*,*N*’,*N*’-Tetraethyl pyridazine 4,5-dicarboxamide 28**

Et_3_N (1.0 mL) was added to a stirred suspension of pyridazine-4,5-dicarboxylic acid (200 mg, 1.2 mmol, 1.0 eq.) and Me_2_NH.HCl (489 mg, 7.5 mmol, 5 eq.) in THF (10 mL) at rt. The resulting solution was stirred at rt for 15 min and then T3P^®^ (2.0 mL of a 50% solution in THF) was added dropwise. The reaction was stirred at rt for 16 h. Then, a saturated solution of NaHCO_3_ (15 mL) and CH_2_Cl_2_ (15 mL) were added and the two layers separated. The aqueous layer was extracted with CH_2_Cl_2_ (2 x 15 mL) and the combined organic layers were dried (MgSO_4_) and concentrated under reduced pressure to give the crude product. Purification by flash column chromatography on silica with 8:2 CH_2_Cl_2_-EtOAc as eluent gave **28** (78 mg, 29%), ^1^H NMR (400 MHz, CD_3_OD) 9.35 (s, 2H), 3.10 (s, 6H), 2.95 (s, 6H); MS (ESI) *m/z* 245 [(M + Na)^+^, 100 ]; HRMS *m/z* calcd for C_10_H_14_N_4_O_2_ (M + Na)^+^ 245.1015, found 245.1019 (+4.1 ppm error).

***N*,*N*,*N*’,*N*’-tetraethyl pyridazine 4,5-dicarboxamide 29**

Et_3_N (0.5 mL) was added to a stirred suspension of pyridazine-4,5-dicarboxylic acid (200 mg, 1.2 mmol, 1.0 eq.) and Et_2_NH (0.5 mL) in THF (10 mL) at rt. The resulting solution was stirred at rt for 15 min and then T3P^®^ (2.0 mL of a 50% solution in THF) was added dropwise. The reaction was stirred at rt for 16 h. Then, a saturated solution of NaHCO_3_ (15 mL) and CH_2_Cl_2_ (15 mL) were added and the two layers separated. The aqueous layer was extracted with CH_2_Cl_2_ (2 x 15 mL) and the combined organic layers were dried (MgSO_4_) and concentrated under reduced pressure to give the crude product. Purification by flash column chromatography on silica with 8:2 CH_2_Cl_2_-EtOAc as eluent gave **29** (154 mg, 46%), ^1^H NMR (400 MHz, CD_3_OD) 9.34 (s, 2H), 3.55 (q., *J* = 7.5 Hz, 4H), 3.22 (q., *J* = 7.5 Hz, 4H), 1.23 (t, *J* = 7.5 Hz, 6H), 1.18 (t, *J* = 7.5 Hz, 6H); MS (ESI) *m/z* 301 [(M + Na)^+^, 100 ]; HRMS *m/z* calcd for C_14_H_22_N_4_O_2_ (M + Na)^+^ 301.1641, found 301.1638 (+3.7 ppm error).

**3.3 NMR spectra of synthesised compounds**

**4-*d*_3_-Methyl pyridazine 4-carboxylate *d*_3_-10**

Figure S7: ^1^H NMR spectrum of 4-*d*_3_-methyl pyridazine 4-carboxylate ***d*_3_-10**.
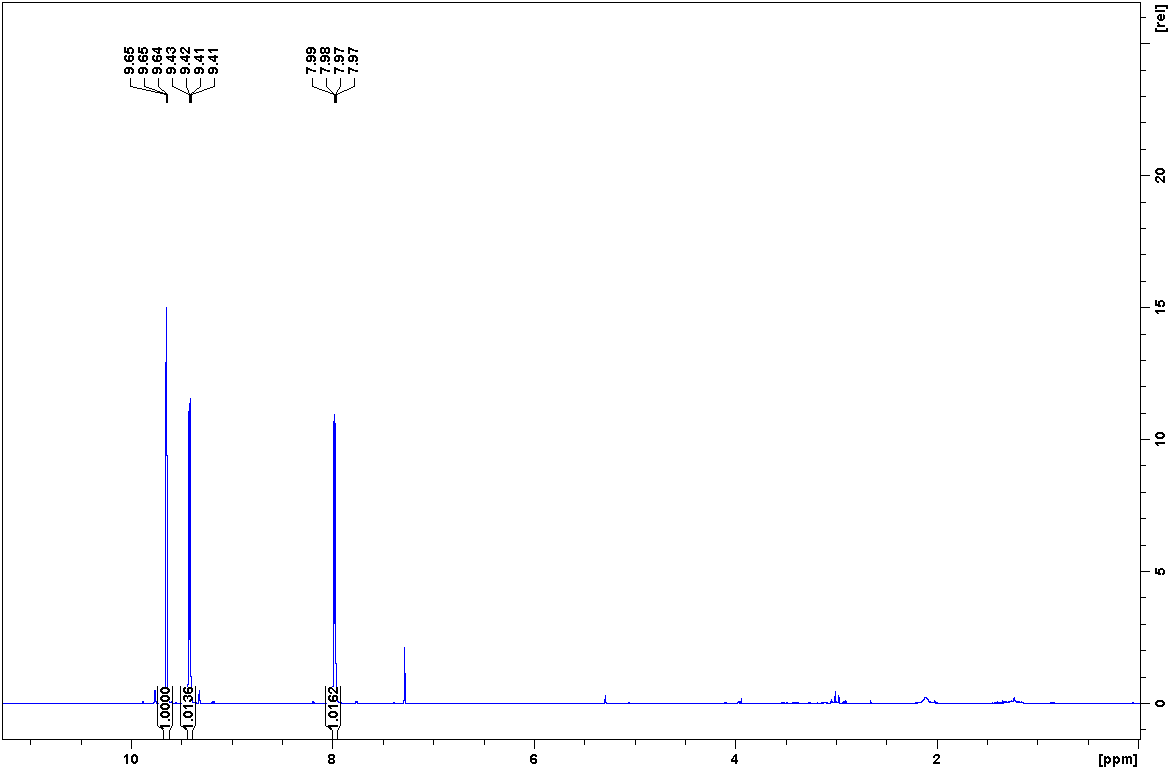


Figure S8: ^13^C{^1^H} NMR spectrum of 4-*d*_3_-methyl pyridazine 4-carboxylate ***d*_3_-10**.


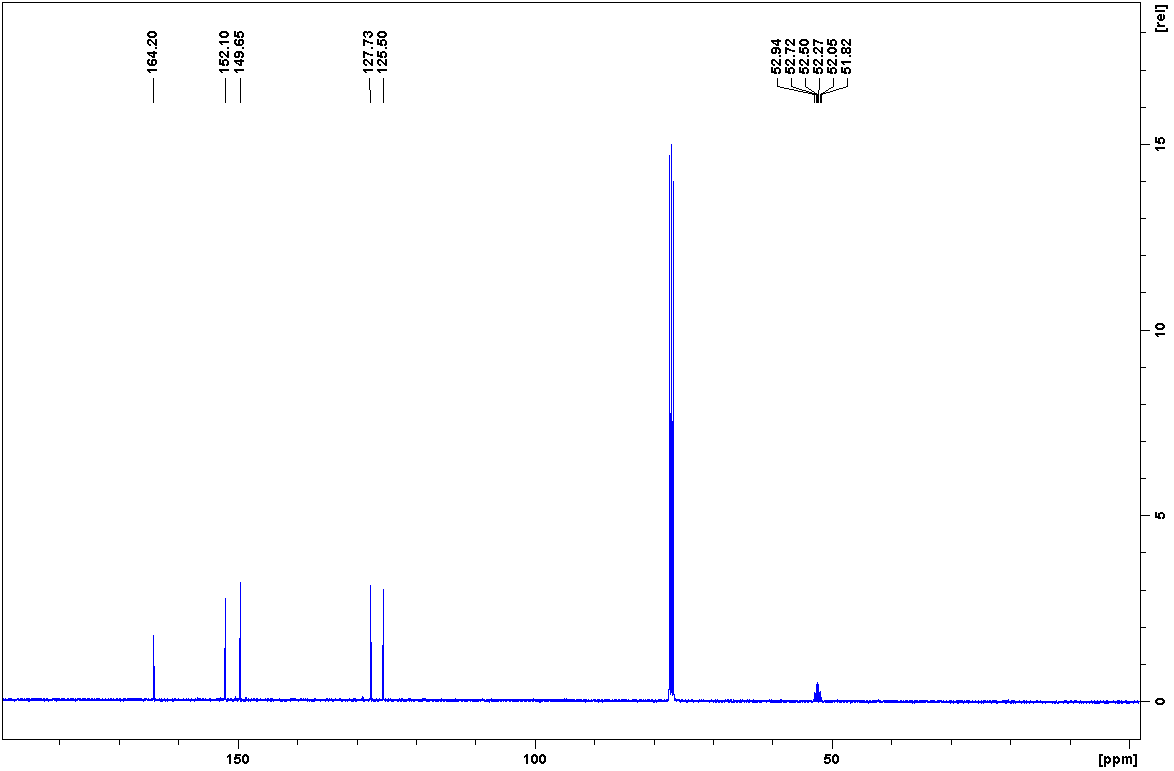


**4,5-Bis(trimethylsilyl) pyridazine 20**

Figure S9: ^1^H NMR spectrum of 4,5-bis(trimethylsilyl) pyridazine **20**.


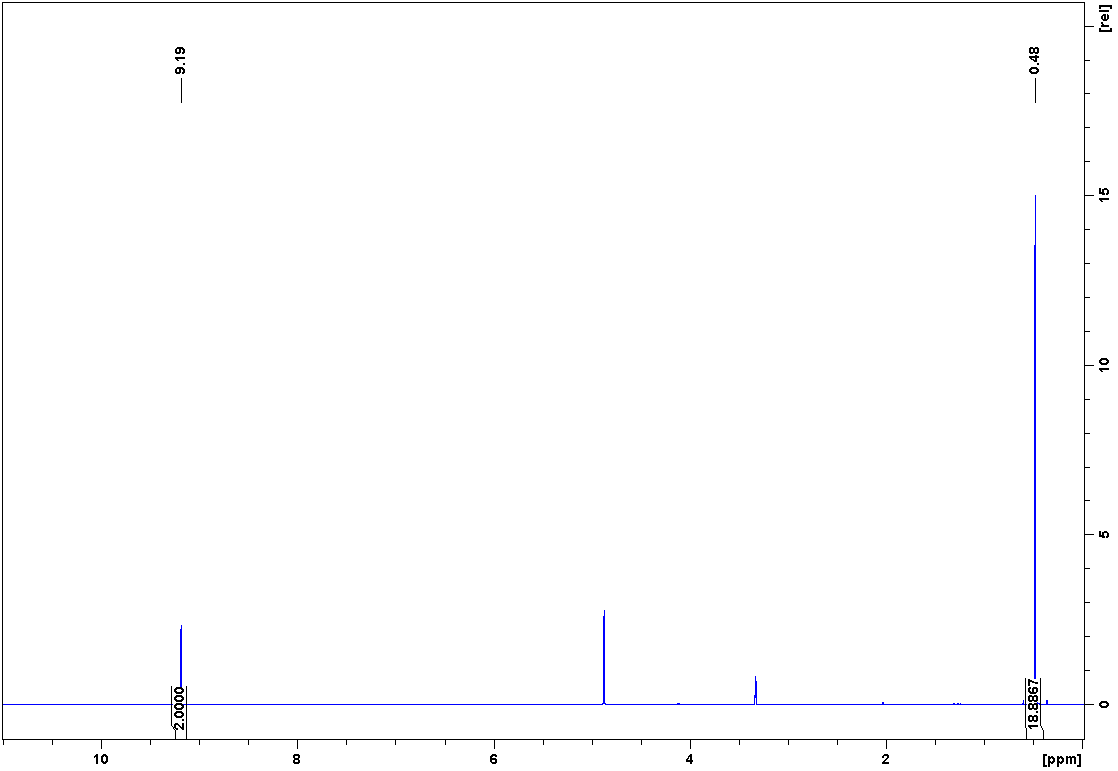


Figure S10: ^13^C{^1^H} NMR spectrum of 4,5-bis(trimethylsilyl) pyridazine **20**.


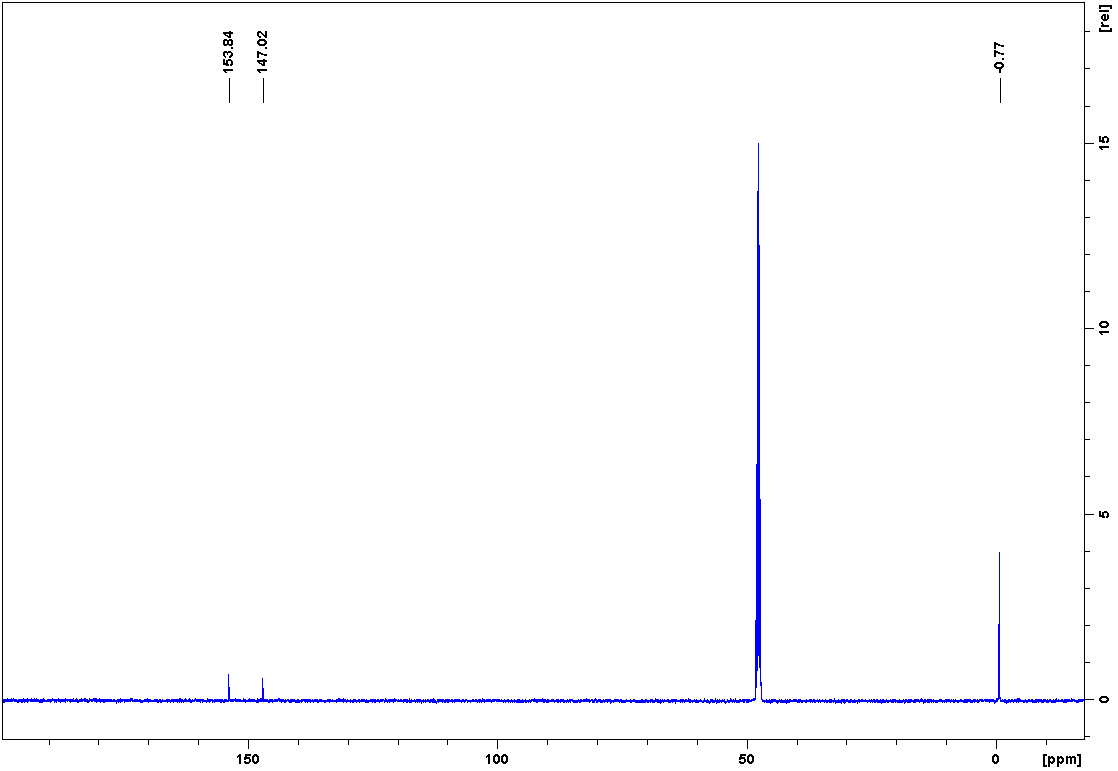


**4,5-Diphenyl pyridazine 21**

Figure S11: ^1^H NMR spectrum of 4,5-diphenyl pyridazine **21**.


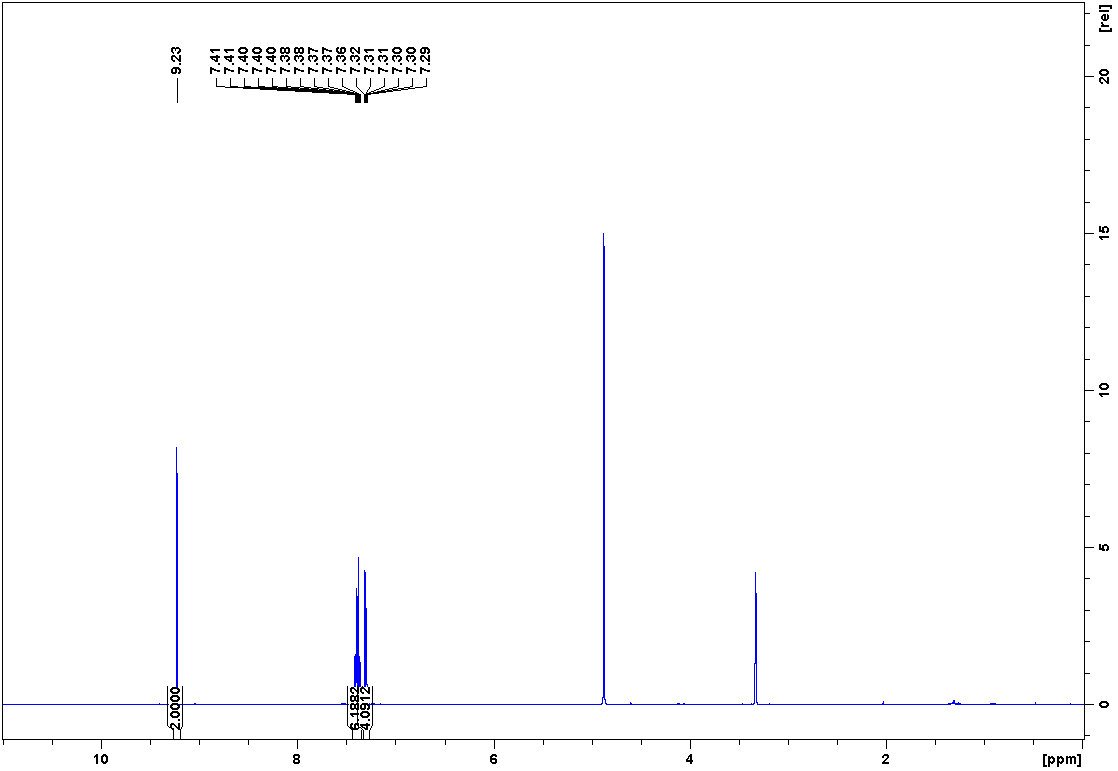


Figure S12: ^13^C{^1^H} NMR spectrum of 4,5-diphenyl pyridazine **21**.


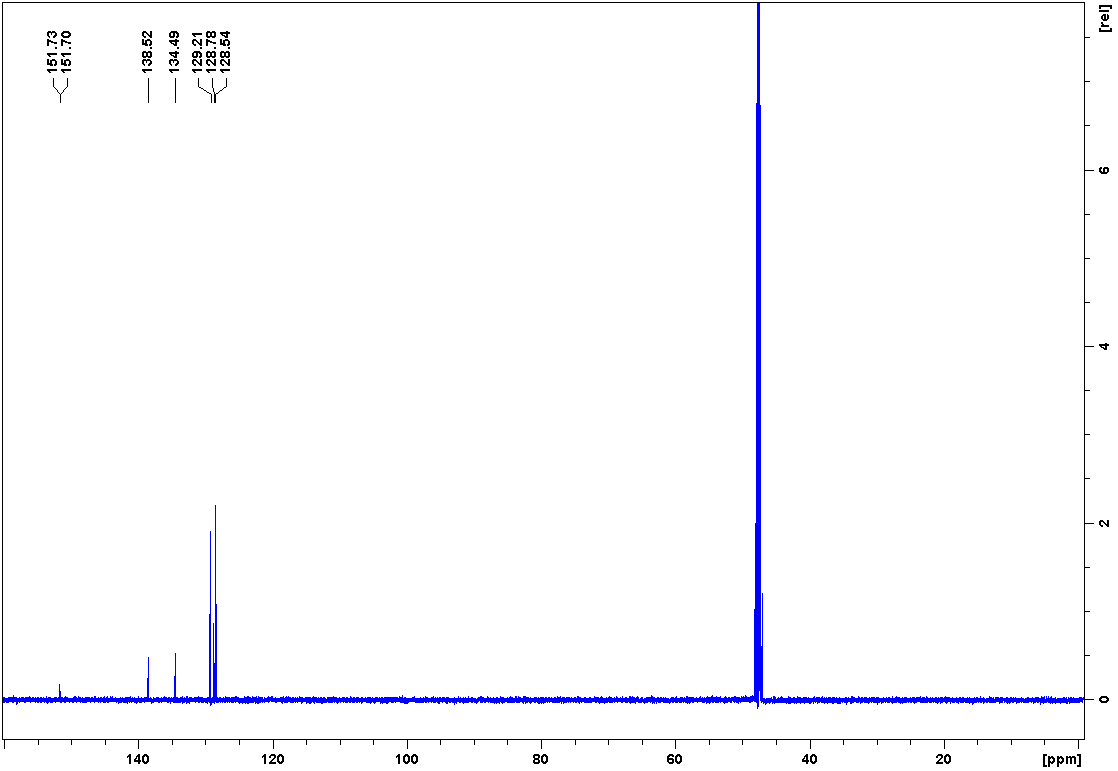


**4,5-Bis(*d*_5_-phenyl) pyridazine *d*_10_-21**

Figure S13: ^1^H NMR spectrum of 4,5-diphenyl pyridazine *d*_10_-**21**.


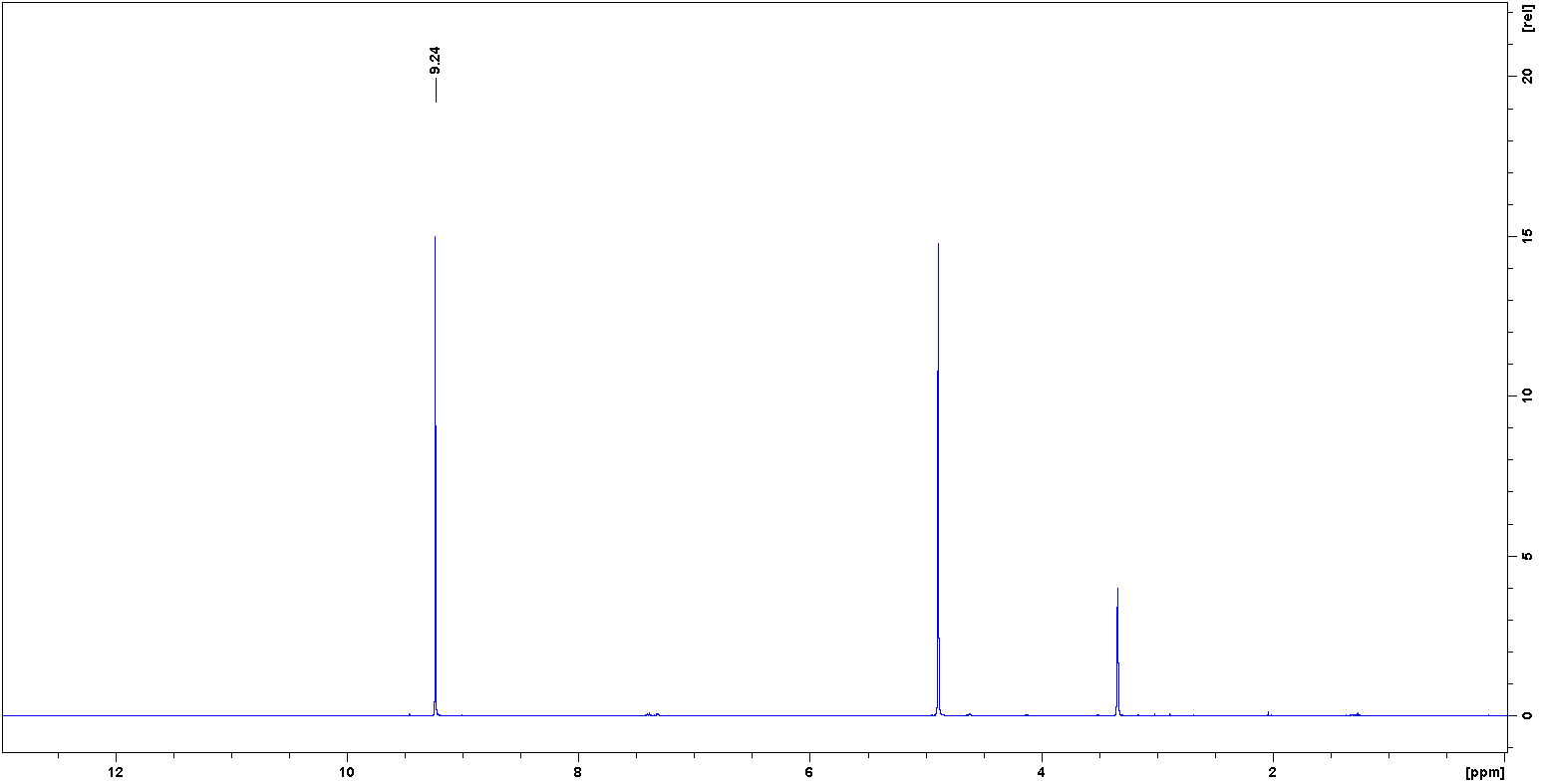

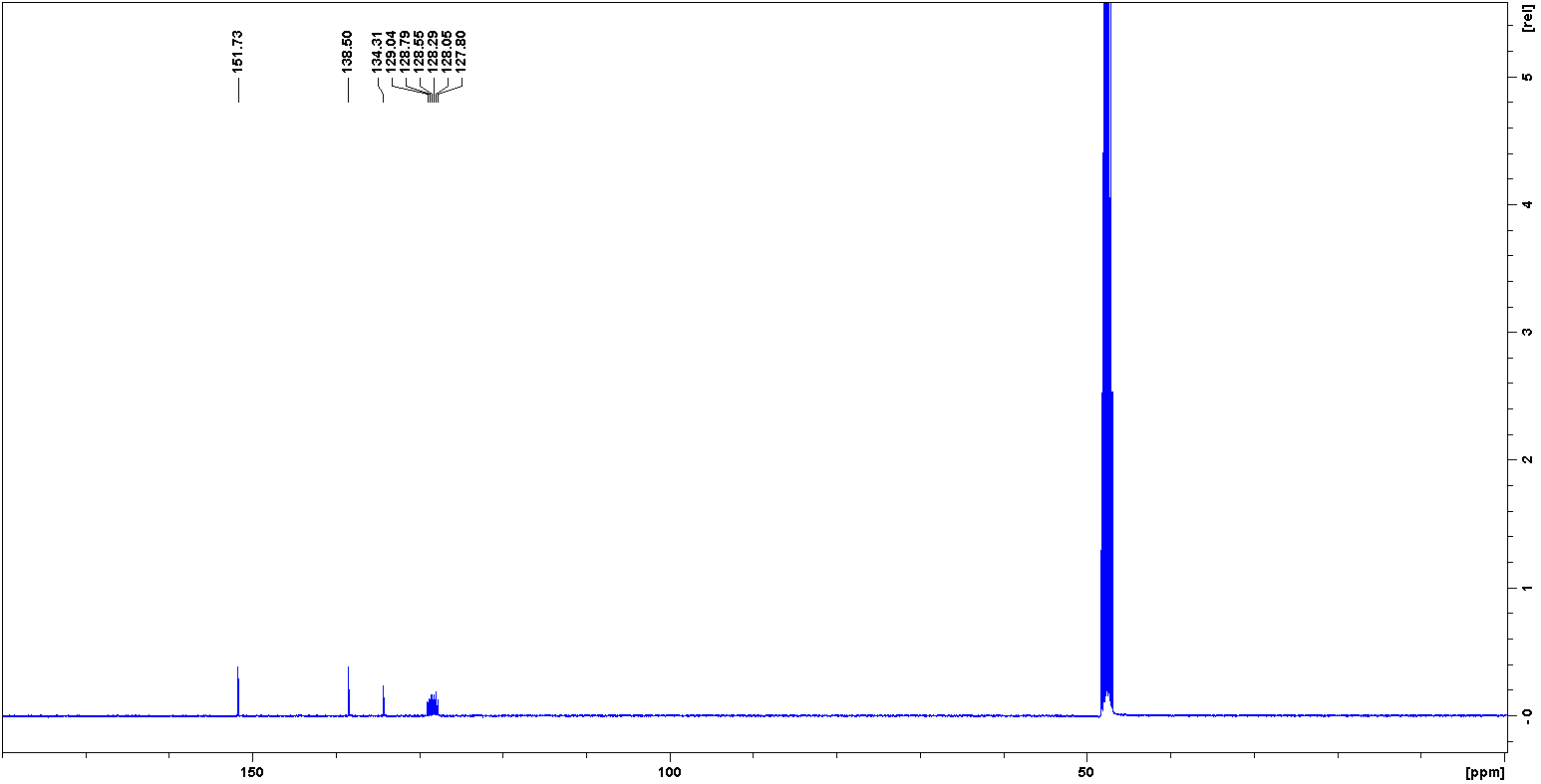


Figure S14: ^13^C{^1^H} NMR spectrum of 4,5-diphenyl pyridazine *d*_10_-**21**.

**4,5-dimethyl pyridazine 4,5-dicarboxylate 23**

Figure S15: ^1^H NMR spectrum of 4,5-dimethyl pyridazine 4,5-dicarboxylate **23**.


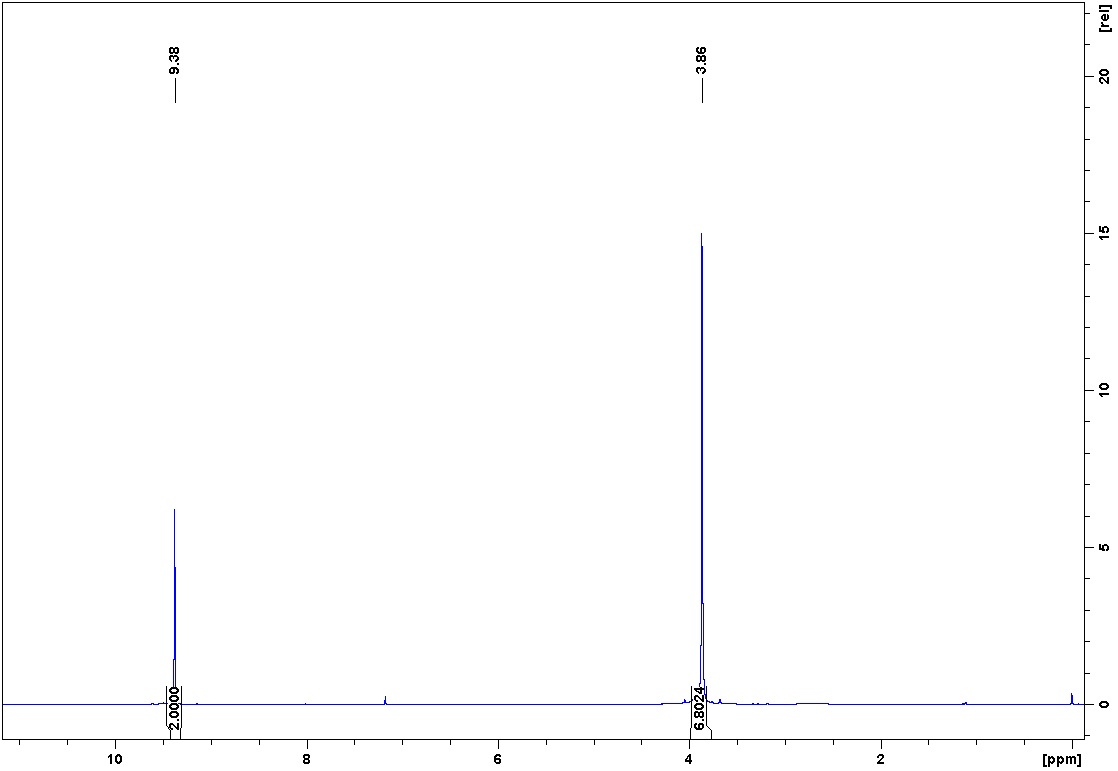


Figure S16: ^13^C{^1^H} NMR spectrum of 4,5-dimethyl pyridazine 4,5-dicarboxylate **23**.


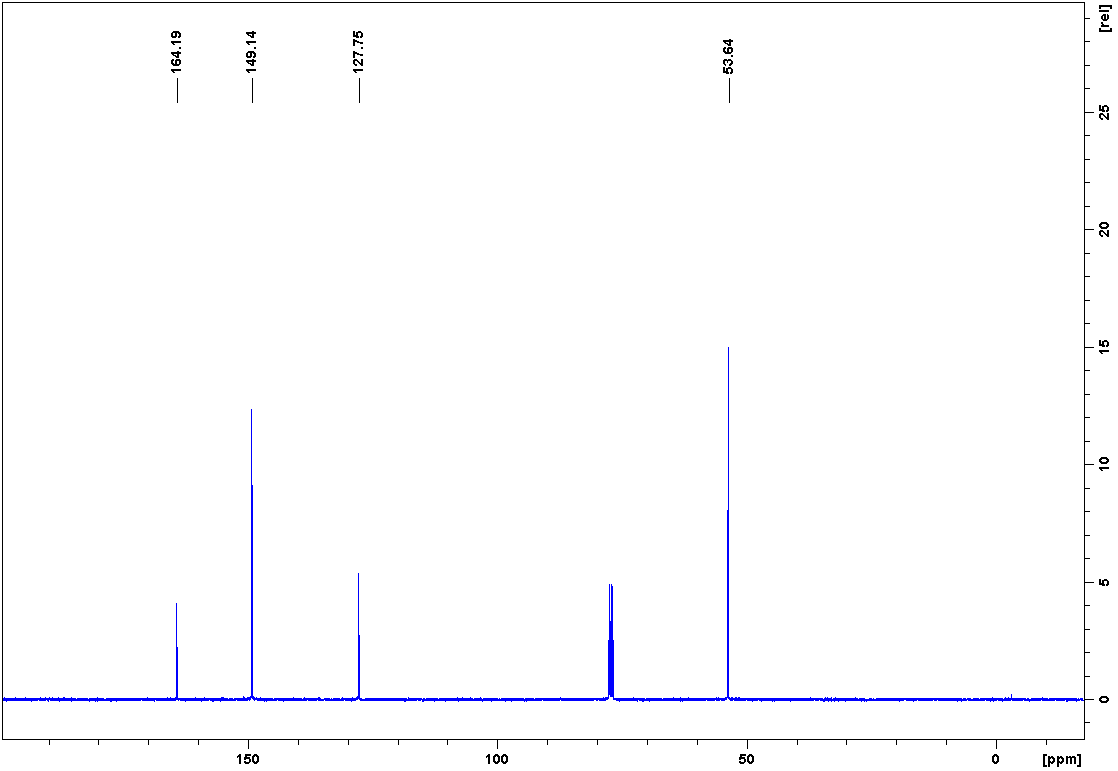


**4,5-Bis(*d*_3_-methyl) pyridazine 4,5-dicarboxylate *d*_6_-23**

Figure S17: ^1^H NMR spectrum of 4,5-bis (*d*_3_-methyl) pyridazine 4,5-dicarboxylate *d*_6_-**23**.
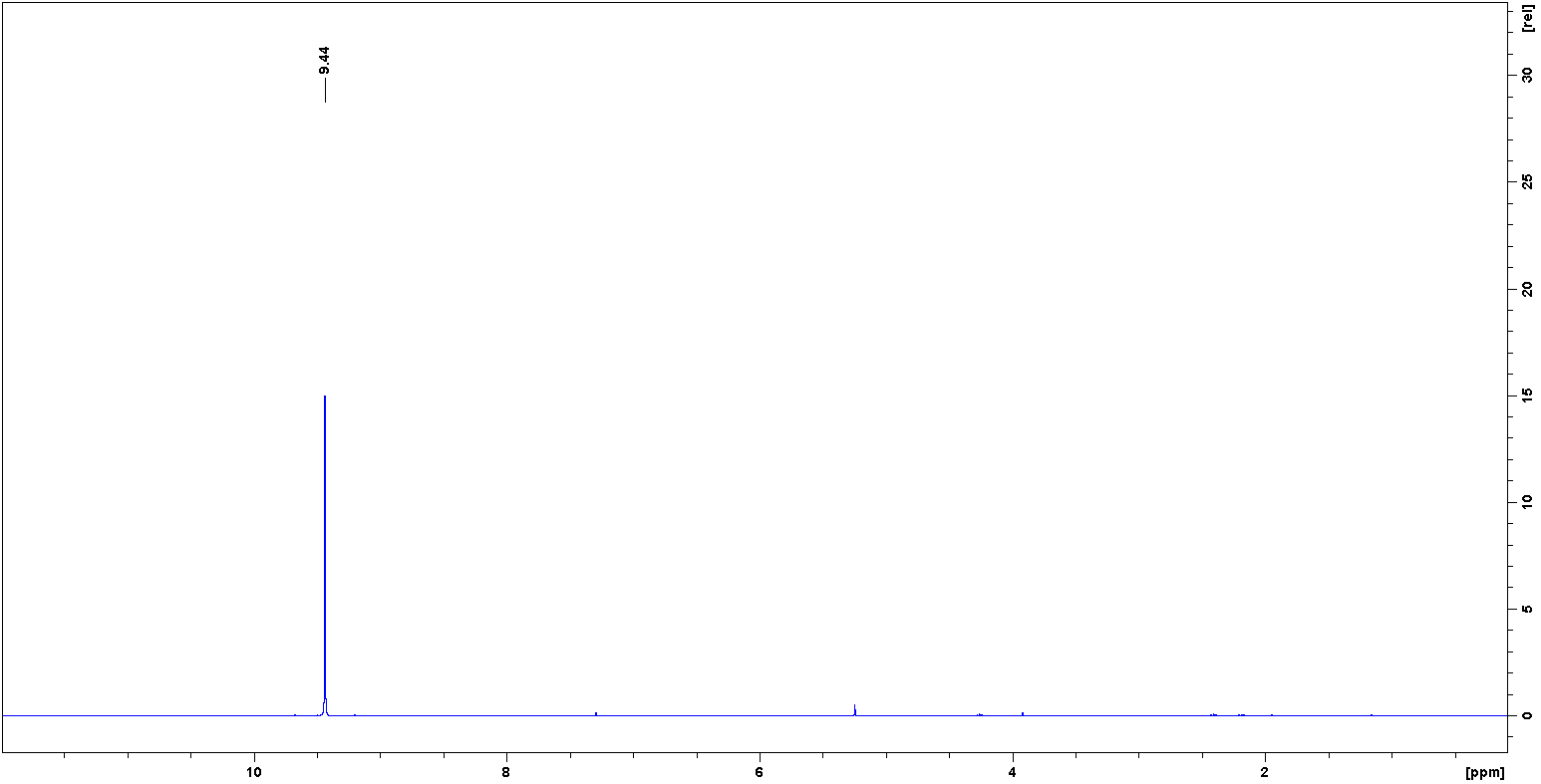


Figure S18: ^13^C{^1^H} NMR spectrum of 4,5-bis (*d*_3_-methyl) pyridazine 4,5-dicarboxylate *d*_6_-**23**.


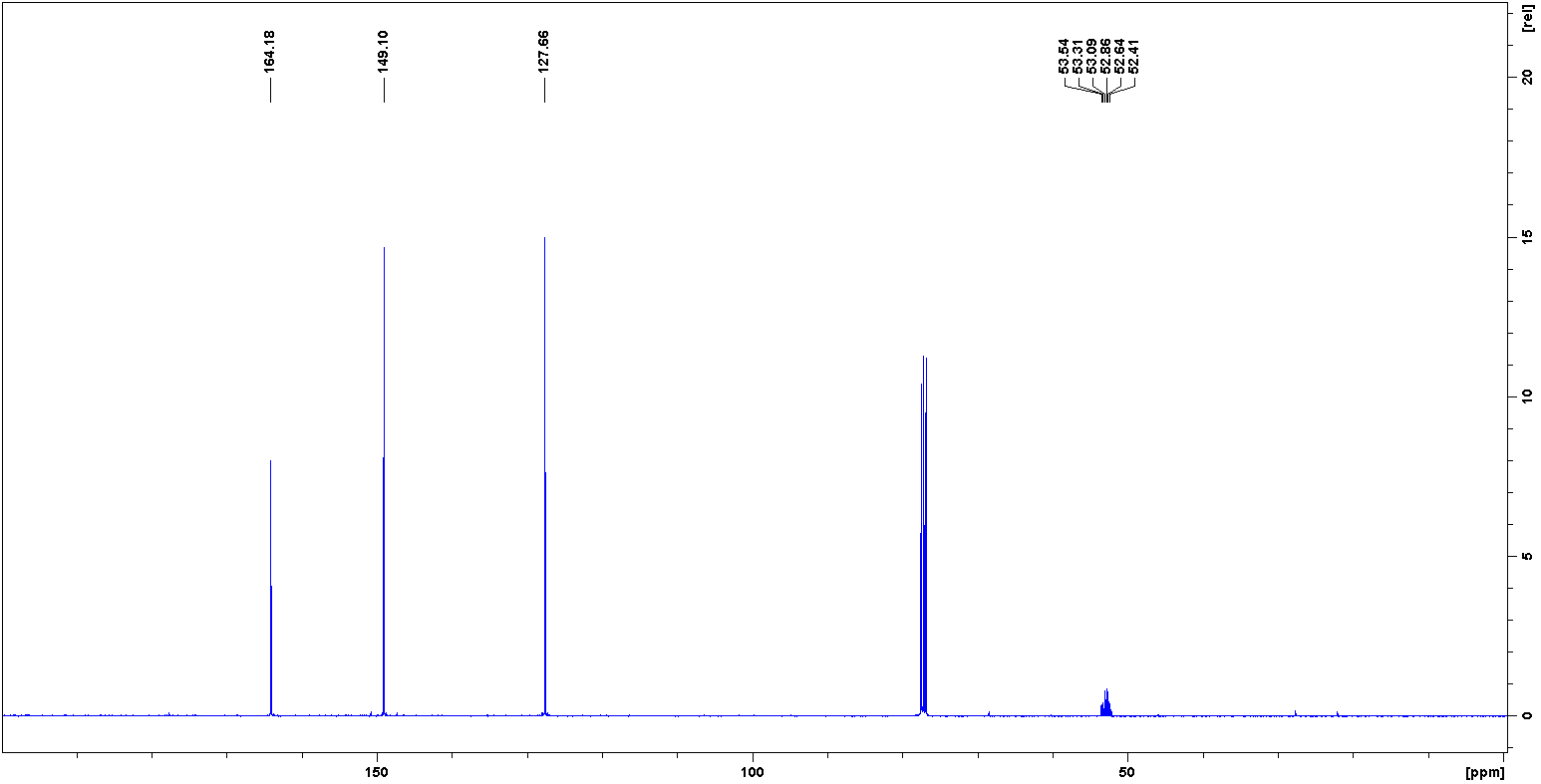


**4-Methyl 5-ethyl pyridazine 4,5-dicarboxylate 26**

Figure S19: ^1^H NMR spectrum of 4-methyl 5-ethyl pyridazine 4,5-dicarboxylate **26**.


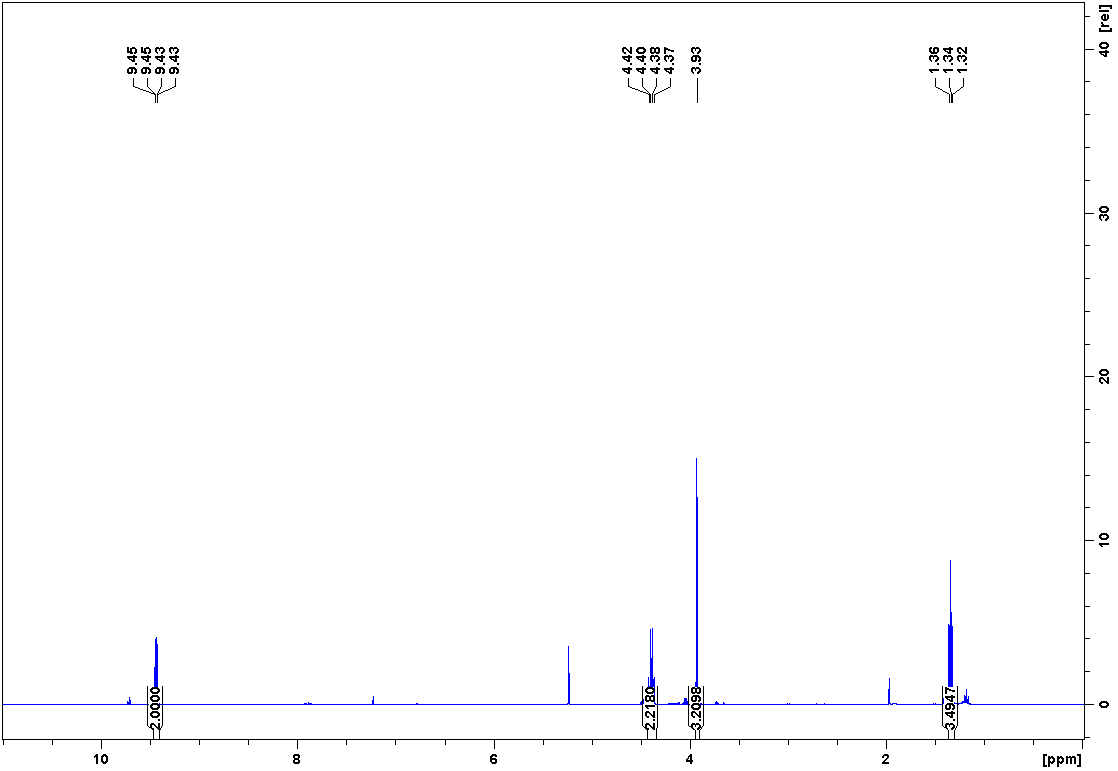


Figure S20: ^13^C{^1^H} NMR spectrum of 4-methyl 5-ethyl pyridazine 4,5-dicarboxylate **26**.


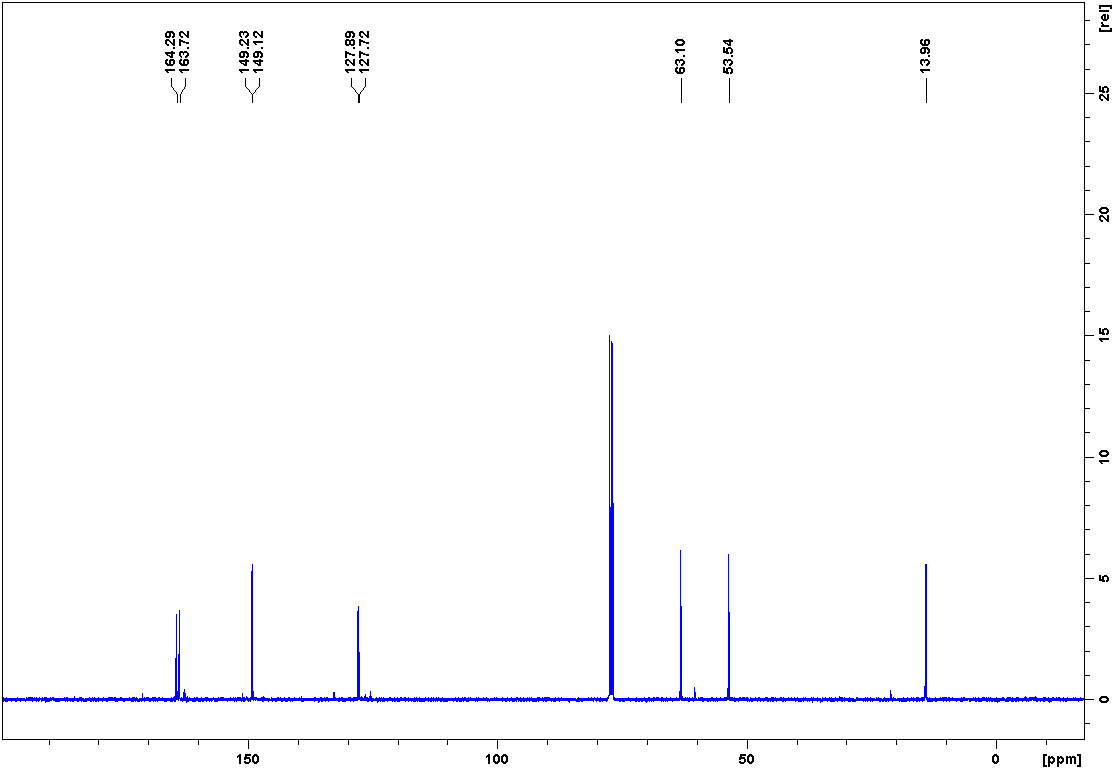


**References**

1. Adams, R. W.; Aguilar, J. A.; Atkinson, K. D.; Cowley, M. J.; Elliott, P. I.; Duckett, S. B.; Green, G. G.; Khazal, I. G.; López-Serrano, J.; Williamson, D. C., Reversible interactions with para-hydrogen enhance NMR sensitivity by polarization transfer. *Science* **2009,** *323* (5922), 1708-1711.

2. Heldmann, D. K.; Sauer, J., Synthesis of metallated (metal = Si, Ge, Sn) pyridazines by cycloaddition of metal substituted alkynes to 1,2,4,5-tetrazine. *Tetrahedron Lett.* **1997,** *38* (33), 5791-5794.

3. Vazquez-Serrano, L. D.; Owens, B. T.; Buriak, J. M., The search for new hydrogenation catalyst motifs based on N-heterocyclic carbene ligands. *Inorg. Chim. Acta* **2006,** *359* (9), 2786-2797.

4. Itai, T.; Kamiya, S., Potential Anti-cancer Agents. XI. Synthesis of 4- and 5-Azidopyridazine 1-Oxide. *CHEMICAL & PHARMACEUTICAL BULLETIN* **1963,** *11* (8), 1059-1064.

5. Ruggiero, A.; Fuchter, M. J.; Kokas, O. J.; Negru, M.; White, A. J. P.; Haycock, P. R.; Hoffman, B. M.; Barrett, A. G. M., A ‘push–pull’ tropylium-fused aminoporphyrazine. *Tetrahedron* **2009,** *65* (47), 9690-9693.
